# Premature mortality trends in 183 countries by cancer type, sex, WHO region, and World Bank income level in 2000–19: a retrospective, cross-sectional, population-based study

**DOI:** 10.1016/S1470-2045(24)00274-2

**Published:** 2024-08

**Authors:** Shilpa S Murthy, Dario Trapani, Bochen Cao, Freddie Bray, Shashanka Murthy, Thomas Peter Kingham, Chandrakanth Are, André M Ilbawi

**Affiliations:** a Department of Surgery, Division of Colon and Rectal Surgery, Yale University School of Medicine, New Haven, CT, USA; b Department of Haematology and Oncology, University of Milan, Milan, Italy; c European Institute of Oncology, IRCCS, Milan, Italy; d Department of Data and Analytics, WHO, Geneva, Switzerland; e Cancer Surveillance, International Agency for Research on Cancer, Lyon, France; f Infectious Disease Institute and Centre of Microbiome Science, Ohio State University, Columbus, OH, USA; g Department of Surgery, Division of Surgical Oncology, Memorial Sloan Kettering Cancer Centre, New York, NY, USA; h Department of Surgery, Division of Surgical Oncology, University of Nebraska Medical Centre, Omaha, NE, USA; i Department of Non-Communicable Diseases, WHO, Geneva, Switzerland

## Abstract

**Background:**

Cancer is a leading cause of mortality worldwide. By 2040, over 30 million new cancers are predicted, with the greatest cancer burden in low-income countries. In 2015, the UN passed the Sustainable Development Goal 3.4 (SDG 3.4) to tackle the rising burden of non-communicable diseases, which calls for a reduction by a third in premature mortality from non-communicable diseases, including cancer, by 2030. However, there is a paucity of data on premature mortality rates by cancer type. In this study, we examine annual rates of change for cancer-specific premature mortality and classify whether countries are on track to reach SDG 3.4 targets.

**Methods:**

This is a retrospective, cross-sectional, population-based study investigating premature mortality trends from 2000–19 using the WHO Global Health Estimates data. All cancers combined and thirteen individual cancers in 183 countries were examined by WHO region, World Bank income level, and sex. The risk of premature mortality was calculated for ages 30–69 years, independent of other competing causes of death, using standard life table methods. The primary objective was to compute average annual rate of change in premature mortality from 2000 to 2019. Secondary objectives assessed whether this annual rate of change would be sufficient to reach SDG 3.4. targets for premature mortality by 2030.

**Findings:**

This study was conducted using data retrieved for the years 2000–19. Premature mortality rates decreased in 138 (75%) of 183 countries across all World Bank income levels and WHO regions, however only eight (4%) countries are likely to meet the SDG 3.4 targets for all cancers combined. Cancers where early detection strategies exist, such as breast and colorectal cancer, have higher declining premature mortality rates in high-income countries (breast cancer 48 [89%] of 54 and colorectal cancer 45 [83%]) than in low-income countries (seven [24%] of 29 and four [14%]). Cancers with primary prevention programmes, such as cervical cancer, have more countries with declining premature mortality rates (high-income countries 50 [93%] of 54 and low-income countries 26 [90%] of 29). Sex-related disparities in premature mortality rates vary across WHO regions, World Bank income groups, and by cancer type.

**Interpretation:**

There is a greater reduction in premature mortality for all cancers combined and for individual cancer types in high-income countries compared with lower-middle-income and low-income countries. However, most countries will not reach the SDG 3.4 target. Cancers with early detection strategies in place, such as breast and colorectal cancers, are performing poorly in premature mortality compared with cancers with primary prevention measures, such as cervical cancer. Investments toward prevention, early detection, and treatment can potentially accelerate declines in premature mortality.

**Funding:**

WHO.

## Introduction

Non-communicable diseases, especially cardiovascular disease, cancer, diabetes, and chronic respiratory diseases are among the leading causes of death globally, accounting for around 74% of deaths worldwide and more than 17 million premature deaths each year.^1^ Cancer is the second leading cause of deaths with more than 9·3 million deaths from non-communicable diseases estimated globally.^1^ By 2050, 35 million new cancers are predicted—a 77% increase from the 20 million cases estimated in 2022—with the greatest burden in lower-middle-income countries (LMICs) and low-income countries (LICs).^2^, ^3^

To tackle the growing burden, the UN and WHO continue to focus on non-communicable diseases as a public health priority.^4^ The 2030 Agenda for Sustainable Development, adopted in 2015, includes goal SDG 3.4 that calls for a reduction by a third in premature mortality, the probability of dying between age 30 and 69 years from four major non-communicable diseases by 2030. Additionally, the World Health Assembly passed resolution 70.12 in 2017 that enabled WHO to prioritise cancer prevention and control programmes, elevating cancer care in the global health agenda.^5^ Compared with other non-communicable diseases, progress in tackling the cancer burden has been slow due to misperceived financial costs and the capacity needed to provide sustainable care.^4^ Multimodal cancer care has traditionally been thought to be resource intensive and costly. Nevertheless, investing in curable cancers and reducing the burden of disease-related disability and productivity loss through comprehensive cancer care, across the cancer continuum, is expected to result in societal and economic gains, as population health is improved.

**Research in context**

**Evidence before this study**

We searched PubMed with the terms “premature mortality AND cancer” for articles published from Aug 1, 2012, to March 23, 2023, in English. Most studies assessed premature mortality from non-communicable diseases including cardiovascular disease, chronic respiratory disease, diabetes, and cancer in combination. Some studies examined premature mortality reductions for non-communicable diseases by individual countries or regions, commonly using WHO Global Health Estimates or Global Burden of Disease. The key message from these studies is that the global burden of cancer is rising, and cardiovascular disease and cancer are now the first or second causes of premature death in most countries and WHO regions. However, no studies evaluated premature mortality by cancer type. The UN prioritised cancer control efforts through Sustainable Development Goal (SDG) 3.4 and WHO cancer resolutions, by focusing on reducing premature cancer mortality to promote a healthy population productive to society and each country's economy. To understand the progress in cancer control there is a crucial need to estimate, track, and monitor changes in premature mortality. However, there is currently no global reference to benchmark and estimate changes in premature mortality by cancer type, World Bank income level, sex, and WHO Region.

**Added value of this study**

Our study provides global estimates on premature mortality, identifying regional and country patterns, that can serve to orient and inform policy toward areas of need, and enable a better understanding of cancer control dynamics, including what key interventions might affect population health. Additionally, we provide the first estimates on whether countries will meet the SDG 3.4 goal of premature mortality reduction; therefore, possibly informing priority areas to be addressed and catalyse efforts toward high-value interventions.

**Implications of all the available evidence**

The global burden of cancer and premature deaths related to malignancies is rising, and at the current rate most countries will not reach SDG 3.4 targets, especially low-income and lower-middle-income countries, where resources and finances are scarce. This study assists policy makers, governments, and clinicians to prioritise high value programmes and policies by cancer type in a given country or WHO region making it contextually relevant locally and globally.

Because WHO and national policy makers consider cancer as a national health priority, contextualising the epidemiological burden of different cancer types is important, especially in LMICs and LICs, where there are competing health priorities. Currently, there is a paucity of data on premature mortality by cancer type. In this study, we examined cancer-specific trends from 2000–19, based on the annual rate of change in premature mortality by country, WHO region, sex, and World Bank income level.

Premature mortality from non-communicable diseases is a standard health indicator used by WHO to monitor progress in reaching SDG 3.4. Premature mortality is defined as the unconditional probability of dying prematurely from a major non-communicable disease (ie, cardiovascular disease, cancer, diabetes, and chronic respiratory disease). Additionally, we quantify whether countries are on track to reach SDG 3.4 by 2030. Our study aims to provide impactful policy solutions for countries seeking to accelerate reductions in cancer premature mortality.

## Methods

### Study design and data sources

For this retrospective, cross-sectional, population-based study we retrieved mortality data by country, year, sex, age, and cancer type from the 2019 WHO Global Health Estimates.^6^ These estimates include 183 member states with a population of 90 000 inhabitants or greater in each country in 2019 and support the monitoring of SDG targets by WHO and the UN (appendix pp 1–3).^7^ Countries were classified according to World Bank income levels (high-income countries [HICs], upper-middle-income countries [UMICs], LMICs, and LICs) and WHO regions (African region, European region, Eastern Mediterranean region, Western Pacific region, South-East Asia region, the region of the Americas, and the world).

We selected all cancers combined and 13 individual cancer types (breast, lung, colorectal, prostate, stomach, liver, cervical, oesophagus, thyroid, bladder, pancreas, leukaemia, and head and neck cancers) based on the Global Cancer incidence and mortality (GLOBOCAN) estimates for 2020 set by the International Agency for Research on Cancer (appendix p 3).^4^, ^8^ This population-level aggregated data was provided by member states of WHO and cleared for publication. Institutional review board or ethical approval is not applicable to this study. Sex data were collected from member states, but race and ethnicity data are not provided to WHO.

### Outcomes

The primary outcome of this study was to examine annual rates of change in premature cancer mortality by cancer type, WHO region, World Bank income level, and sex for the years 2000–19.^4^, ^8^ For the secondary outcome, we determined whether a country's annual rate of change in premature mortality was on track to meet an annual target rate of change by 2030 that would result in premature mortality reduction by a third, stratified by cancer type. We identified the number of countries and WHO regions that are on track to meet SDG 3.4.

### Statistical analysis

Premature mortality estimates between the ages of 30 and 69 years are derived from age-specific death rates using life table methods, assuming an absence of other competing causes of death (appendix p 4).^7^, ^10^, ^11^ This was a secondary analysis of published WHO estimates using a pre-defined SDG indicator (ie, premature mortality) and the standard calculation methods have been previously published by WHO. Potential biases can stem from the underlying data and have been discussed extensively elsewhere.^12^, ^13^

To calculate premature mortality estimates for 2030 we applied the standardised WHO approach.^10^, ^11^, ^13^ To benchmark historical trends with SDG 3.4, we estimated the reduction in national mortality rates required to meet the target for a specific cancer type or for all cancers combined, assuming the percentage reduction is constant across age groups and causes addressed by the non-communicable diseases target. To reach the target of a reduction by a third in premature mortality, the simplest scenario would be reducing mortality rates from each individual type of cancer and the other three major non-communicable diseases at the same rate for all 5-year age groups (ages 30–69 years; appendix p 4).

We calculated the average annual rate of change in premature mortality from 2000–19 using the following formula:^7^, ^13^

$$\text{Average annual rate}=\ln\left(\frac{\text{Premature}{\text{mortality}}_{2019}}{\text{Premature}{\text{mortality}}_{2000}}\right)/(2019-2020)$$

We similarly calculated a target average annual rate of change in premature mortality for 2015–30. If premature mortality estimates for 2000 or 2015 (or both) were missing, we used the nearest years to those to develop the estimates.

To determine whether countries were on target to reach SDG 3.4, we divided the average annual rate of change in premature mortality for 2000–19 by the target average annual rate of change in premature mortality for 2015–30. If the resulting country's rate ratio was more than 0·85–1·10 then their annual rate of change in premature mortality had a higher possibility to meet SDG 3.4 by 2030, the ratios were categorised as shown in the appendix (p 4). We use the rate ratios to visualise the degree of progress towards reaching SDG 3.4. All analysis was performed using R, STATA SE (version 15), and Microsoft Excel.

### Role of funding source

The funder of the study had no role in study design, data collection, data analysis, data interpretation, or writing of the report.

## Results

**Figure 1:**
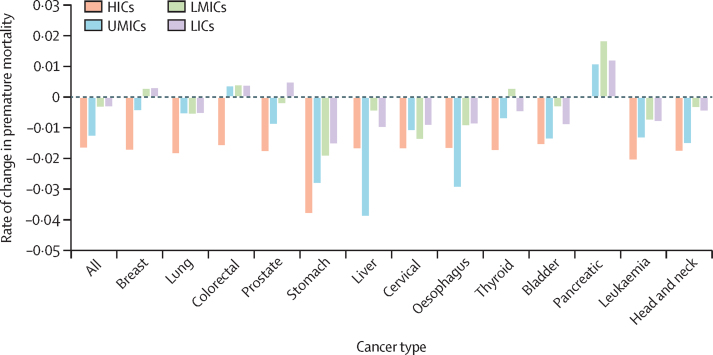
Annual rate of change in premature mortality in 2000–19 for cancers by World Bank income level HICs=high-income countries. LICs=low-income countries. LMICs=lower-middle-income countries. UMICs=upper-middle-income countries.

**Figure 2:**
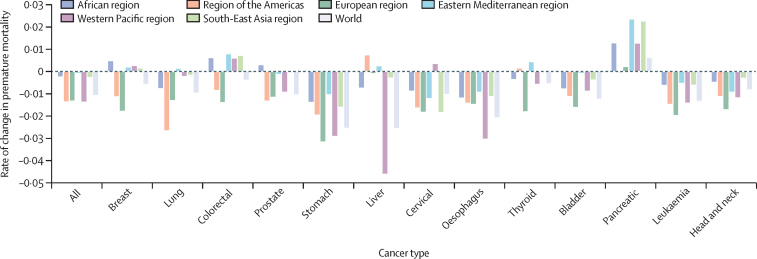
Annual rate of change in premature mortality in 2000–19 for cancers by WHO region

This study was done using data retrieved for the years 2000–19. Premature mortality rates declined in most World Bank income categories and WHO regions (Figure 1, Figure 2). Premature mortality rates declined for countries at all World Bank income levels for all cancers combined and for lung, stomach, liver, cervical, oesophageal, bladder, leukaemia, and head and neck cancers (figure 1). Premature mortality rates declined for all WHO regions for all cancers combined, stomach, oesophageal, bladder, leukaemia, and head and neck cancers (figure 2). Pancreatic cancer had rising premature mortality rates in all WHO regions, except for the region of the Americas.

**Figure 3:**
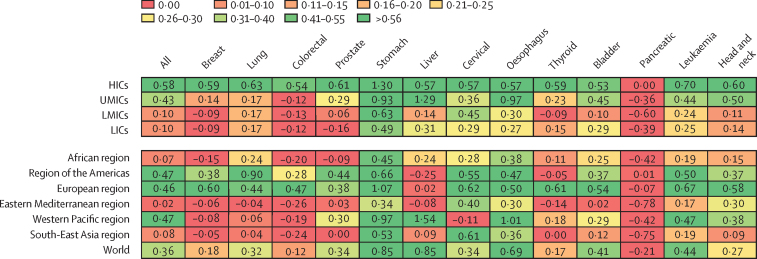
Rate ratios for cancers by World Bank income level and WHO region Rate ratio is the average annual rate of change in premature mortality for 2000–19 divided by the target average annual rate of change in premature mortality from 2015–30. The rate ratios correspond to gradient colours and were categorised as shown in the appendix (p 4). If the resulting country's rate ratio was more than 0·85–1·10 then their annual rate of change in premature mortality had a higher possibility to meet SDG 3.4 by 2030. HICs=high-income countries. LICs=low-income countries. LMICs=lower-middle-income countries. UMICs=upper-middle-income countries.

For most countries the rates are not declining fast enough to reach SDG 3.4 targets (figure 3). No WHO region or World Bank income level category will reach SDG 3.4 targets for all cancers combined. Stomach cancer is on track to reach the SDG 3.4 target in HICs and in UMICs, and in the European and Western Pacific WHO regions. Other cancers on track to reach SDG 3.4 are liver cancer and oesophageal cancer in UMICs and Western Pacific region, and lung cancer in the region of the Americas (figure 3).

Overall, premature mortality rates declined for 138 (75%) of 183 countries, but only eight (4%) are on track to meet SDG 3.4 targets for all cancers combined (Table 1, Table 2). When analysing by sex, premature mortality rates for all cancers combined declined in all World Bank income categories and WHO regions except for females in the Eastern Mediterranean region (appendix p 5).

**Table 1:** Number of countries with declining premature mortality rates in 2000–19 by cancer type, World Bank income level, and WHO region

	**All**	**Breast**	**Lung**	**Colorectal**	**Prostate**	**Stomach**	**Liver**	**Cervical**	**Oesophagus**	**Thyroid**	**Bladder**	**Pancreatic**	**Leukaemia**	**Head and neck**
Total (n=183)	138 (75%)	94 (51%)	115 (63%)	77 (42%)	93 (51%)	172 (94%)	112 (61%)	166 (91%)	135 (74%)	112 (61%)	130 (71%)	40 (22%)	146 (80%)	143 (78%)
HICs (n=54)	51 (94%)	48 (89%)	51 (94%)	45 (83%)	44 (81%)	54 (100%)	29 (54%)	50 (93%)	43 (80%)	45 (83%)	44 (81%)	23 (43%)	51 (94%)	50 (93%)
UMICs (n=51)	38 (75%)	24 (47%)	33 (65%)	19 (37%)	25 (49%)	49 (96%)	34 (67%)	44 (86%)	41 (80%)	28 (55%)	30 (59%)	11 (22%)	43 (84%)	43 (84%)
LMICs (n=49)	31 (63%)	15 (31%)	21 (43%)	8 (16%)	14 (29%)	43 (88%)	26 (53%)	46 (94%)	32 (65%)	23 (47%)	30 (61%)	3 (6%)	35 (71%)	32 (65%)
LICs (n=29)	18 (62%)	7 (24%)	10 (34%)	4 (14%)	10 (34%)	26 (90%)	23 (79%)	26 (90%)	19 (66%)	16 (55%)	26 (90%)	3 (10%)	17 (59%)	18 (62%)
African region (n=47)	28 (60%)	11 (23%)	19 (40%)	9 (19%)	13 (28%)	40 (85%)	34 (72%)	41 (87%)	28 (60%)	28 (60%)	38 (81%)	5 (11%)	29 (62%)	28 (60%)
Region of the Americas (n=33)	25 (76%)	14 (42%)	23 (70%)	9 (27%)	21 (64%)	31 (94%)	17 (52%)	29 (88%)	28 (85%)	14 (42%)	19 (58%)	8 (24%)	24 (73%)	26 (79%)
European region (n=50)	47 (94%)	46 (92%)	44 (88%)	39 (78%)	35 (70%)	49 (98%)	26 (52%)	46 (92%)	35 (70%)	40 (80%)	38 (76%)	15 (30%)	47 (94%)	45 (90%)
Eastern Mediterranean region (n=21)	13 (62%)	9 (43%)	10 (48%)	8 (38%)	10 (48%)	21 (100%)	14 (67%)	20 (95%)	17 (81%)	9 (43%)	17 (81%)	4 (19%)	17 (81%)	19 (90%)
Western Pacific region (n=21)	17 (81%)	7 (33%)	15 (71%)	10 (48%)	10 (48%)	21 (100%)	14 (67%)	19 (90%)	18 (86%)	16 (76%)	12 (57%)	6 (29%)	19 (90%)	17 (81%)
South-East Asia region (n=11)	8 (73%)	7 (64%)	4 (36%)	2 (18%)	4 (36%)	10 (91%)	7 (64%)	11 (100%)	9 (82%)	5 (45%)	6 (55%)	2 (18%)	10 (91%)	8 (73%)

The percentage is based on the denominators of the total number of countries in that specific WHO region or World Bank income classification. HICs=high-income countries. LICs=low-income countries. LMICs=lower-middle-income countries. UMICs=upper-middle-income countries.

**Table 2:** Number of countries on track to meet SDG 3.4

	**All**	**Breast**	**Lung**	**Colorectal**	**Prostate**	**Stomach**	**Liver**	**Cervical**	**Oesophagus**	**Thyroid**	**Bladder**	**Pancreatic**	**Leukaemia**	**Head and neck**
Total (n=183)	8 (4%)	20 (11%)	18 (10%)	10 (5%)	20 (11%)	64 (35%)	15 (8%)	32 (17%)	26 (14%)	25 (14%)	22 (12%)	2 (1%)	20 (11%)	24 (13%)
HICs (n=54)	4 (7%)	18 (33%)	10 (19%)	9 (17%)	17 (31%)	36 (67%)	7 (13%)	16 (30%)	12 (22%)	20 (37%)	13 (24%)	2 (4%)	17 (31%)	17 (31%)
UMICs (n=51)	3 (6%)	2 (4%)	4 (8%)	0	2 (4%)	16 (31%)	6 (12%)	11 (22%)	9 (18%)	5 (10%)	6 (12%)	0	2 (4%)	6 (12%)
LMICs (n=49)	0	1 (2%)	4 (8·2%)	1 (2%)	1 (2%)	10 (20%)	2 (4%)	4 (8%)	3 (6%)	0	3 (6%)	0	1 (2%)	1 (2%)
LICs (n=29)	0	0	0	0	0	2 (7%)	0	1 (3%)	2 (7%)	0	0	0	0	0
African region (n=47)	0	0	0	0	0	6 (13%)	2 (4%)	3 (6%)	3 (6%)	0	2 (4%)	0	0	2 (4%)
Region of the Americas (n=33)	0	1 (3%)	4 (12%)	0	4 (12%)	11 (33%)	2 (6%)	8 (24%)	9 (27%)	4 (12%)	4 (12%)	1 (3%)	0	5 (15%)
European region (n=50)	3 (6%)	15 (30%)	8 (16%)	6 (12%)	14 (28%)	30 (60%)	4 (8%)	9 (18%)	7 (14%)	15 (30%)	10 (20%)	1 (2%)	15 (30%)	8 (16%)
Eastern Mediterranean region (n=21)	2 (10%)	3 (14%)	2 (10%)	1 (5%)	2 (10%)	6 (29%)	2 (10%)	5 (24%)	3 (14%)	2 (10%)	2 (10%)	0	3 (14%)	4 (19%)
Western Pacific region (n=21)	2 (10%)	0	2 (10%)	3 (14%)	0	8 (38%)	5 (24%)	4 (19%)	4 (19%)	3 (14%)	2 (10%)	0	1 (5%)	4 (19%)
South-East Asia region (n=11)	1 (9%)	2 (18%)	2 (18%)	0	0	3 (27%)	0	3 (27%)	0	1 (9%)	2 (18%)	0	1 (9%)	1 (9%)

Countries fall into the high and very high categories for rate ratio calculations (appendix p 4). HICs=high-income countries. LICs=low-income countries. LMICs=lower-middle-income countries. UMICs=upper-middle-income countries.

Premature mortality rates declined for breast cancer in 94 (51%) of 183 countries; however, even in HICs only 18 (33%) of 54 are on trajectories to reach SDG 3.4 (Table 1, Table 2). Increasing rates of premature mortality occurred for males with breast cancer in UMICs and in the Western Pacific region, whereas decreasing rates were seen for females with breast cancer in UMICs (appendix p 6).

Even though lung cancer premature mortality rates were decreasing in most countries, only 18 (10%) are on track to reach targets (Table 1, Table 2). For females with lung cancer, premature mortality rates increased in all income groups except in HICs, the region of the Americas, and the Western Pacific region (appendix p 7).

Ten (5%) of 183 countries are on a trajectory to reach SDG 3.4 for colorectal cancer (table 2). Colorectal cancer premature mortality rates are rising for males and females in LMICs and LICs but decreasing for females in UMICs (appendix p 8). 93 (51%) countries have declining trends in prostate cancer, yet only 20 (11%) are on track to reach SDG 3.4 (Table 1, Table 2).

For stomach cancer, 64 (35%) countries appear on track to meet SDG 3.4 targets and both females and males with stomach cancer had declining rates of premature mortality by both WHO region and World Bank income level (table 2; appendix p 9). For liver cancer, only 15 (8%) countries will reach SDG targets (table 2; appendix p 10). Premature mortality rates have declined for males and females with liver cancer in all World Bank income level countries but increased for males in the region of the Americas and in the Eastern Mediterranean region, and for females in the Eastern Mediterranean region (appendix p 10). Premature mortality declines occurred in 166 (91%) countries for cervical cancer, but only 32 (17%) countries will reach SDG 3.4 targets (Table 1, Table 2).

Similar to stomach cancer, oesophageal cancer premature mortality rates decreased for all WHO regions and World Bank Income levels; this finding was also true for males and females (appendix p 11). Premature mortality rates declined for thyroid cancer, however only 25 (14%) countries will reach SDG 3.4. Rates of premature mortality increased for males with thyroid cancer in UMICs, LMICs, the region of the Americas, Eastern Mediterranean region, Western Pacific region, and South-East Asia region (appendix p 12).

For bladder cancer, premature mortality rates have declined for males and females by World Bank income level and WHO region (table 1; appendix p 13); only 22 (12%) of countries are likely to reach the SDG 3.4 target.

For pancreatic cancer, 40 (22%) countries had declining premature mortality rates (table 1), and only two (1%) are predicted to reach SDG 3.4 (table 2; Antigua and Barbuda, and Ireland). Males with pancreatic cancer in HICs and the region of the Americas had declining rates of premature mortality; this pattern was not evident in females (appendix p 14).

Leukaemia premature mortality rates have declined in 146 (80%) countries, however, only 20 (11%) countries are likely to reach the SDG target for leukaemia. Both females and males with leukaemia had declining rates of premature mortality for all WHO regions and World Bank income levels (table 2; appendix p 15).

Premature mortality rates declined for male and females with head and neck cancer for all World Bank income levels and WHO regions, including in 143 (78%) countries (table 1, appendix p 16). However, only 24 (13%) countries are predicted to reach the SDG 3.4 target for head and neck cancer (table 2).

## Discussion

To our knowledge, this is the first study to assess worldwide trends in premature mortality by cancer type, WHO region, World Bank income level, and sex. Our results highlight that although premature mortality rates are declining, they are not on track to reach SDG 3.4 targets by 2030 for most countries. Some premature mortality reductions have been observed for multiple cancers, including breast, colorectal, and cervical cancers; however, SDG 3.4 targets will rarely be met. This is also seen in HICs, LMICs, and LICs, even though primary prevention is available in HICs for cervical cancer and screening and early diagnosis programmes are also available for cervical, breast, and colorectal cancers. These early detection programmes are good value for money as they improve population health through cost-effective interventions capable of reducing cancer mortality—and are included in WHO Best buys, making them priority investment cancers for all countries.^14^

Premature mortality from non-communicable diseases is a standard indicator chosen by WHO because it excludes confounding across countries and over time due to differences or changes in mortality rates for other competing causes.^15^ We aimed to assess premature mortality of cancer based on observed data, which itself already reflects the impact of competing causes. There is a paucity of premature mortality data by cancer type; however, our findings are consistent with previous estimates—eg, in the *Women, power, and cancer: a* Lancet *Commission*, breast, lung, colorectal, and cervical cancer were the leading causes of premature mortality for women, which is concurrent with our findings.^16^

Compared with cancers that have primary prevention strategies (eg, hepatitis B vaccination to prevent liver cancer and human papillomavirus [HPV] vaccination to prevent cervical cancer) and tobacco risk reduction programmes (for lung, bladder, and head and neck cancers), those amenable to early detection (eg, breast and colorectal cancer) are not on track to reach SDG 3.4 by 2030, despite available curative treatments. These premature mortality trends are probably due to the rising incidence of breast cancer since 2007, attributed to the obesity epidemic and an increase in screening in HICs.^3^, ^4^

Premature mortality for colorectal cancer is high compared with other cancers, which can be attributed to rising colorectal cancer incidence rates and ineffective screening and early diagnosis programmes.^3^, ^4^ Incidence in colorectal cancer doubled worldwide from 1990 to 2019 and is highest in Australia, New Zealand, and North America; however, premature mortality rates in these countries declined from 2000 to 2019.^3^, ^4^, ^17^ This trend is likely because many HICs have well developed early detection programmes that use population-based colonoscopy to detect early stage cancers.^3^, ^4^

Colorectal cancer premature mortality rates declined for females in UMICs, the region of the Americas, Western Pacific, and the European regions, warranting further research to better understand these trends. In transitioning economies, such as eastern Europe and South America, colorectal cancer incidence and premature mortality rates are rising. This shift might be due to transitions in socioeconomic development and epidemiological shifts as well as increasing obesity rates, cigarette smoking, heavy alcohol consumption, and red meat consumption.^3^, ^17^, ^18^ HICs and the European region are not on track to reach SDG 3.4 for colorectal cancer. This finding might be due to the rising incidence rates of colorectal cancer in patients younger than 50 years—a population that is not typically included in screening guidelines—in countries such as the USA, where obesity and other related risk factors are rising.^3^, ^17^, ^18^

There has been limited progress in early detection programmes in resource-constrained health systems. The cost of implementing and maintaining these programmes, including equipment (such as colonoscopy and mammography) and associated training for the workforce, remains a challenge for LICs. However, these upfront costs could mitigate the long-term effects that premature death has on a country's workforce and economy. The WHO Global Breast Cancer Initiative is supporting progress by collaborating with governments to build capacity for early detection and strengthen health-care systems to improve outcomes for patients with breast cancer.^19^

Primary prevention for lung cancer (ie, tobacco control) likely accounts for the large number of countries with declining premature mortality rates.^20^ Sex-based trends in our study show that premature mortality rates for females with lung cancer are rising in all World Bank income levels, except in HICs, mirroring increased smoking habits in women since 1970s.^3^ Other factors include climate change, environmental pollution, radon exposure, and biological factors that need to be investigated further.^3^, ^20^ Programmes, such as WHO MPOWER, provide guidelines for countries to implement and manage tobacco control.^3^, ^20^ The declining premature mortality trends for lung cancer in some countries can probably be attributed to the success of these programmes and lung cancer screening. Lung screening with low-dose CT for individuals at high risk is a resource intensive and expensive method. Screening efforts are feasible on a population level in HICs but are unlikely to be achievable in LMICs and LICs.^21^

Primary prevention and early detection strategies such as HPV vaccination and Papanicolaou [PAP] smears might account for most countries with declining premature mortality rates for cervical cancer. Since cervical cancer is mostly caused by infection with HPV, vaccination and screening programmes can prevent most cases. If detected at early stages, treatment methods such as surgery, radiotherapy, and chemotherapy can help to improve survival and lead to cure. However, our study shows that none of the WHO regions will reach SDG 3.4 targets for cervical cancer, highlighting the need to accelerate HPV vaccination, early detection programmes, and treatment of invasive cancers. In LMICs and LICs, the incidence and mortality rates of cervical cancer are 2–3 times those in HICs and survival is poor due to fragmented medical and public health systems.^3^, ^4^, ^22^ For example, in the Western Pacific region, which has less favourable trends in premature mortality and only four of 37 countries in the region have national HPV immunisation programmes.^23^ Screening participation rates are low, with only four countries reaching 70% immunisation coverage. Primary care facilities face challenges in terms of funding, infrastructure, and workforce in order to implement screening services effectively. Insufficient community awareness and knowledge on cervical cancer and screening contributes to low uptake and clear referral pathways. In August, 2020, the World Health Assembly adopted the Global Strategy for cervical cancer elimination, which will hopefully accelerate the reduction of premature mortality rates to reach SDG 3.4 goals.^24^

Declining incidence rates for stomach and oesophageal cancers might explain the large number of countries with decreasing premature mortality rates. SDG 3.4 targets for both cancers are likely to be met in several countries. For stomach cancer, this decrease in premature mortality might be due to improved preservation of foods via refrigeration and declining rates of *Helicobacter pylori* infection due to its discovery and treatment. The lag in improvement in LMICs and LICs is related to rising risk factors, such as consumption of tobacco and alcohol, obesity rates, and Epstein–Barr virus infection, which is prevalent in African countries.^3^, ^4^, ^25^ In many LMICs and LICs, access to treatment for *H pylori*, refrigeration due to an absence of electricity and clean water, and early detection via endoscopy are limited due to absence of appropriate infrastructure as well as fragmented and constrained health systems.

Biology of disease might also contribute to differences in premature mortality rates; adenocarcinoma is the predominant type of oesophageal cancer in many HICs (linked to obesity rates) as are the rising gastric cardia-based cancers;^26^ whereas in Asia and Africa, squamous cell carcinoma remains the predominant histological type.^3^, ^26^ Screening programmes exist in countries with a high burden of stomach cancer, including Japan and South Korea; however, endoscopy can be resource intensive and might not be feasible in resource-constrained countries. Prevention through lifestyle modifications, testing for *H pylori*, and WHO MPOWER programmes, could make the largest difference in accelerating the decline in premature mortality rates.

Liver cancer incidence rates are highest in socially and economically transitioning countries and common in eastern Asia and Africa. Risk factors include chronic infection with hepatitis B and C virus, aflatoxin-contaminated foods, heavy alcohol consumption, obesity, type 2 diabetes, and smoking.^3^, ^4^, ^27^ Liver cancer is harder to treat and cure than gastrointestinal cancers, such as colorectal cancer. Still, premature mortality rates for liver cancer seem to perform better than colorectal cancer in terms of premature mortality rate reductions. This finding might be a consequence of population-based immunisation programmes for hepatitis B and anti-hepatitis C medications, which are a part of the WHO Global Hepatitis Programme that aims to reduce new hepatitis infections by 90% and deaths by 65% between 2016 and 2030.^28^ Our premature mortality estimates showed that the Western Pacific region is set to reach SDG 3.4, which might be attributed to the success of hepatitis B vaccination coverage by countries in this region (eg, vaccination coverage reached 99% in China, 95% in Australia, 96% in Mongolia, and 94% in Viet Nam).^29^ The Western Pacific region includes 20 countries with national hepatitis action plans and ten scaled up hepatitis services at the primary health-care level.^29^ HICs lag behind UMICs and the Western Pacific region due to the rise of non-alcoholic fatty liver disease caused by metabolic syndrome that is driven by non-viral factors. In the USA, liver cancer incidence is increasing at a high rate, which is attributed to metabolic syndrome and an ageing population.^27^ Although the USA has more than 91% coverage for hepatitis vaccination, absence of liver cancer screening protocols in patients with non-alcoholic fatty liver disease contributes to late diagnosis and presentation with higher stage liver cancers. Non-alcoholic fatty liver disease is projected to increase to 56% between 2016 and 2030 in countries such as Japan, the UK, and the USA.^27^

Prostate cancer is detectable at early stages using prostate-specific antigen and a digital rectal exam. If localised disease is diagnosed early, life expectancy can be 99% for 10 years.^30^ However, population-based screening has been controversial. The PLCO screening trial^31^ showed an increase in the rate of prostate cancer diagnosis, but no improvement in cancer-specific mortality with prostate-specific antigen screening after 13 years of follow-up. The ERSPC study also showed increased incidence of prostate cancer in screened individuals, but a 20% reduction in cancer-specific mortality and did not affect overall survival.^32^ So, population-based screening programmes have not been rolled out in countries with high socioeconomic indexes due to the risk of overdiagnosis and overtreatment of indolent prostate cancers. The factors contributing to poor premature mortality rates are multifactorial and biology of disease might play a role. For example, men of Asian descent living in Asia have a lower risk of prostate cancer than White men living in the USA.^33^ Men of African or Caribbean descent living in the USA have two times higher relative risk of aggressive prostate cancer than White men.^34^

The thyroid cancer incidence rate is three times higher for females than for males, which might partly explain the decreasing premature mortality rates for females; however, further research is needed to explain why premature mortality is higher for men. Since the 1980s there has been a rise in the diagnosis of papillary thyroid cancers—often subclinical and indolent in nature.^35^ This increase is due to newer diagnostic imaging technologies, such as CT and ultrasound, and has led to overdiagnosis, especially for women in Denmark and the UK.^35^ In India, in areas with better access to health care, overdiagnosis accounted for 51% of thyroid cancer diagnoses and 74% of cases in those aged younger than 35 years compared with the rate of diagnosis in communities where health care was less accessible.^36^ Because resources in LMICs and LICs are scarce, overdiagnosis and overtreatment can be harmful and divert resources towards low-value care and expose patients to an avoidable risk of harm.

In the Eastern Mediterranean region, there is a high incidence of bladder cancer and diagnosis is often made at advanced stages, making the cancer harder to treat.^37^ Similar to lung cancer, late prioritisation and implementation of programmes that control tobacco smoking, a risk factor for bladder cancer, in the Eastern Mediterranean region might affect the reduction of premature mortality rates.^38^, ^39^ Other risk factors include schistosomiasis—endemic in Africa and Eastern Mediterranean—which might contribute to poor reductions in premature mortality in these regions.^40^

Pancreatic cancer has shown the worst premature mortality trends compared with all other cancers in our study. This finding can be attributed to the aggressive tumour biology, challenges in early detection, and treatment, as pancreatic cancer is diagnosed more commonly at advanced stages and the available treatment options provide limited survival benefits. In HICs, incidence rates are higher than in LMICs and LICs probably due to access to diagnostic technology and there is evidence showing that pancreatic cancer deaths will surpass those of breast cancer in European countries by 2025.^41^ High rates of obesity, diabetes, and alcohol consumption also contribute to pancreatic cancer deaths and public health prevention measures to tackle these issues can possibly improve premature mortality outcomes. However, scientific progress to further elucidate the biology of disease, development of technologies for early detection, and better treatments are going to be key factors to improve survival from pancreatic cancer.

Epidemiological trends in premature mortality for adult leukaemia are similar to global patterns of access to treatment. In the absence of prevention and early detection programmes, investments in sustainable treatments are a priority for leukaemia. Acute lymphoblastic leukaemia treatment requires health system capacity for appropriate diagnosis, treatment, and supportive care and chronic myeloid leukaemia can be treated with imatinib.^42^ Almost 90% of patients who have received imatinib have a life expectancy nearly as long as adults unaffected by leukaemia.^43^ Poor outcomes for people with acute lymphoblastic and chronic myeloid leukaemia in LMICs and LICs are associated with financial hardships due to unaffordability of treatment, higher rates of treatment abandonment due to costs and side-effects, and an elevated risk of treatment-related mortality.

Head and neck cancers are driven by viral (eg, HPV and Epstein–Barr virus) and lifestyle risk factors (eg, alcohol and tobacco use) and are usually reported as a group of diseases due to the low prevalence of individual tumour types. Premature mortality from head and neck cancer appears to be declining, which could be credited to HPV vaccination and WHO MPOWER programmes. LMICs and LICs might not reach SDG 3.4 targets for head and neck cancers because risk factors, such as betel nut chewing in India and Central Asia and tobacco smoking, are persistent, along with suboptimal access to curative treatments.^44^ A global strategy on oral health was adopted at the World Health Assembly, WHA74·5 resolution.^45^, ^46^ This declaration will provide economic and programmatic assistance for governments to develop oral health policies, a health-care workforce, and new research agendas. This type of commitment can accelerate the decline in premature mortality for head and neck cancers.

One of the limitations of this study is related to data quality from the WHO Global Health Estimates. Countries in LMICs and LICs might not have well-functioning death registration systems (eg, some countries in Africa rely on verbal autopsy studies with variation in the instruments used and insufficient validation). In countries where death registration systems are poor, all-cause mortality estimates rely on census data, survey data, and verbal autopsy which might not be as accurate as in countries with well-functioning systems. Another limitation is the comparison of premature mortality of cancer with other non-communicable diseases, which was beyond the scope of this study. Two strengths of our study findings include the use of global data and global generalisability.

Cancer is a complex set of individual diseases with variations in risk factors, biology of disease, detection, and treatment.^3^ This study aims to assist policy makers, governments, and clinicians to prioritise high value programmes, interventions, and policies by cancer type. Our assessment of premature mortality burden of cancer will help with the creation of local and global cancer policies and hopefully be a step towards planning and creating comprehensive national cancer control programmes that might reduce premature mortality rates worldwide.

### Contributors

### Data sharing

All data used in this study are WHO data and can be accessed online at Global health estimates and Leading causes of death. Data was given to WHO by countries that are member states and cleared before publication.

## Declaration of interests

We declare no competing interests.
