# Supplementary material for: Premature mortality trends in 183 countries by cancer type, sex, WHO region, and World Bank income level in 2000–19: a retrospective, cross-sectional, population-based study
Source: Lancet Oncol. 2024 Aug;25(8):969–78. doi: 10.1016/S1470-2045(24)00274-2 (PMC11329430; doi:10.1016/S1470-2045(24)00274-2)
Supplement: Supplementary appendix [file mmc1.pdf]

THE LANCET
Oncology

Supplementary appendix

This appendix formed part of the original submission and has been peer reviewed. We post it as supplied by the authors.

Supplement to: Murthy SS, Trapani D, Cao B, et al. Premature mortality trends in 183 countries by cancer type, sex, WHO region, and World Bank income level in 2000–19: a retrospective, cross-sectional, population-based study. *Lancet Oncol* 2024; published online July 1. https://doi.org/10.1016/S1470-2045(24)00274-2.

Appendix

Supplement 1: Countries By World Bank Classification

Our World Bank (WB) definitions have been taken based on the 2019-2020 classifications. Which at that time WB classified low-income countries with gross national income (GNI) per capita of $1035 or less; low middle-income economies have GNI per capita between $1036 and $4045 in 2019; Upper Middle-Income economies GNI per capita between $4,046 and $12,535; High-Income Economics are those with GNI per capita of $12,536 or more. (Based on 2019-2020 WB definitions)

We had a total of 183 countries were analysed: 54 high-income countries (HIC), 51 upper middle-income countries (UMIC), 49 low middle-income countries (LMIC), and 29 low-income countries (LIC) .There were 47 countries in the African region (AFRO), 33 in Americas region (AMRO), 50 in Europe (EURO), 21 in Eastern Mediterranean region (EMRO), 21 in Western Pacific region (WPRO), and 11 countries in South-East Asia (SEARO)

https://datahelpdesk.worldbank.org/knowledgebase/articles/906519-world-bank-country-and-lending-groups

High Income Countries (54 countries): United Arab Emirates, Antigua and Barbuda, Australia, Austria, Belgium, Bahrain, Bahamas, Barbados, Brunei Darussalam, Canada, Switzerland, Chile, Cyprus, Czechia, Germany, Denmark, Spain, Estonia, Finland, France, United Kingdom, Greece, Croatia, Hungry, Ireland, Iceland, Israel, Italy, Japan, Republic of Korea, Kuwait, Lithuania, Luxembourg, Latvia, Malta, Mauritius, Netherlands, Norway, New Zealand, Oman, Panama, Poland, Portugal, Qatar, Romania, Saudi Arabia, Singapore, Slovakia, Slovenia, Sweden, Seychelles, Trinidad and Tobago, Uruguay, United States of America

Upper Middle-Income Countries (51 countries): Albania, Argentina, Armenia, Azerbaijan, Bulgaria, Bosnia and Herzegovina, Belarus, Belize, Brazil, Botswana, China, Colombia, Costa Rica, Cuba, Dominican Republic, Ecuador, Fiji, Gabon, Georgia, Equatorial Guinea, Grenada, Guatemala, Guyana, Indonesia, Iran (Islamic Republic of), Iraq, Jamaica, Jordan, Kazakhstan, Lebanon, Libya, Saint Lucia, Maldives, Mexico, North Macedonia, Montenegro, Malaysia, Namibia, Peru, Paraguay, Russian Federation, Serbia, Suriname, Thailand, Turkmenistan, Tonga, Turkey, Saint Vincent and the Grenadines, Venezuela (Bolivarian Republic of), Samoa, South Africa

Low- and Middle-Income Countries(49 countries): Angola, Benin, Bangladesh, Bolivia (Plurinational State of), Bhutan, Cote d'Ivoire, Cameroon, Congo, Comoros, Cabo Verde, Dijibouti, Algeria, Egypt, Micronesia (Federated States of), Ghana, Honduras, India, Kenya, Kyrgyzstan, Cambodia, Kiribati, Lao People's Democratic Republic, Sri Lanka, Lesotho, Morocco, Republic of Moldova, Myanmar, Mongolia, Mauritania, Nigeria, Nicaragua, Nepal, Pakistan, Philippines, Papua New Guinea, Senegal, Solomon Islands, El Salvador, Sao Tome and Principe, Eswatini, Timor-Leste, Tunisia, United Republic of Tanzania, Ukraine, Uzbekistan, Vietnam, Vanuatu, Zambia, Zimbabwe

Low Income Countries (29 countries): Afghanistan, Burundi, Burkina Faso, Central African Republic, Democratic Republic of the Congo, Eritrea, Ethopia, Guinea, Gambia, Guinea-Bissau, Haiti, Liberia, Madagascar, Mali, Mozambique, Malawi, Niger, Democratic People's Republic of Korea, Rwanda, Sudan, Sierra Leone, Somalia, South Sudan, Syrian Arab Republic, Chad, Togo, Tajikistan, Uganda, Yemen

WHO Regions

African Region (AFRO)	Region of the Americas (AMRO)	European Region (EURO)	Eastern Mediterranean Region (EMRO)	Western Pacific Region (WPRO)	South-East Asia Region (SEARO)
Angola	Argentina	Albania	Afghanistan	Australia	Bangladesh
Burundi	Antigua and Barbuda	Armenia	United Arab Emirates	Brunei Darussalam	Bhutan
Benin	Bahamas	Austria	Bahrain	China	Indonesia
Burkina Faso	Belize	Azerbaijan	Djibouti	Fiji	India
Botswana	Bolivia	Belgium	Egypt	Micronesia, Federated States of	Sri Lanka
Central African Republic	Brazil	Bulgaria	Iran, Islamic Republic of	Japan	Maldives
Cote d'Ivoire	Barbados	Bosnia and Herzegovina	Iraq	Cambodia	Myanmar
Cameroon	Canada	Belarus	Jordan	Kiribati	Nepal
Democratic Republic of the Congo	Chile	Switzerland	Kuwait	Korea, Republic of	Democratic People's Republic of Korea
Comoros	Colombia	Cyprus	Lebanon	Lao People's Democratic Republic	Thailand
Cabo Verde	Costa Rica	Czech Republic	Libya	Mongolia	Timor-Leste
Algeria	Cuba	Germany	Morocco	Malaysia	
Eritrea	Dominican Republic	Denmark	Oman	New Zealand	
Ethiopia	Ecuador	Spain	Pakistan	Philippines	
Gabon	Grenada	Estonia	Qatar	Papua New Guinea	
Ghana	Guatemala	Finland	Saudi Arabia	Singapore	
Gambia	Guyana	France	Sudan	Solomon Islands	
Guinea-Bissau	Honduras	United Kingdom	Somalia	Viet Nam	
Equatorial Guinea	Haiti	Georgia	Syrian Arab Republic	Vanuatu	
Kenya	Jamaica	Greece	Tunisia	Samoa	
Liberia	Saint Lucia	Croatia	Yemen		
Lesotho	Mexico	Hungary			
Madagascar	Nicaragua	Ireland			
Mali	Panama	Iceland			
Mozambique	Peru	Israel			
Mauritania	Paraguay	Italy			
Mauritius	El Salvador	Kazakhstan			
Malawi	Suriname	Kyrgyzstan			
Namibia	Trinidad and Tobago	Lithuania			
Niger	Uruguay	Luxembourg			
Nigeria	United States of America	Latvia			
Rwanda	Saint Vincent and the Grenadine	Moldova, Republic of			

Senegal	Venezuela	North Macedonia			
Sierra Leone		Malta			
South Sudan		Montenegro			
Sao Tome and Principe		Netherlands			
Eswatini		Norway			
Seychelles		Poland			
Chad		Portugal			
Togo		Romania			
Tonga		Russian Federation			
Tanzania, United Republic of		Serbia			
Uganda		Slovakia			
South Africa		Slovenia			
Zambia		Sweden			
Zimbabwe		Tajikistan			
		Turkmenistan			
		Turkey			
		Ukraine			
		Uzbekistan			

Cancer Classification ICD-10 codes

Cancers that are included in our data set are classified according to the International Classification of Disease version 10. We ranked and included cancers according to the estimated incident number of cases reported by the International Agency for Research on Cancer, Global Cancer Observatory data from 2020 available on their website below.

International Classification Disease (ICD)		Categories and Abbreviations Utilized in Study
C00-97	All cancers	All cancers (All)
C50	Breast	Breast
C33-34	Lung, Trachea, Bronchus	Lung
C18-21	Colon, Rectum, Anus	Colorectal (CRC)
C61	Prostate	Prostate
C16	Stomach	Stomach
C22	Liver and intrahepatic bile ducts	Liver
C53	Cervix Uteri	Cervical
C73	Thyroid	Thyroid
C15	Oesophagus	Esophagus
C82-86, C96	Non-Hodgkin lymphoma	Non-Hodgkin lymphoma (NHL)
C54	Corpus uteri	Uterine
C67	Bladder	Bladder
C00-C13, C32	Hypopharynx, Oropharynx, Lip, Oral cavity, Salivary glands, Nasopharynx, Larynx	Head and Neck (H&N)
C64-65	Kidney and renal pelvis	Kidney
C25	Pancreas	Pancreas
C56	Ovary	Ovarian
C91-95	Leukemia	Leukemia

https://gco.iarc.fr/today/online-analysis-table?v=2018&mode=cancer&mode_population=continents&population=900&populations=900&key=asr&sex=0&cancer=39&type=0&statistic=5&prevalence=0&population_group=0&ages_group%5B%5D=6&ages_group%5B%5D=13&group_cancer=1&include_nmsc=1&include_nmsc_other=1

Premature Mortality Calculations

Premature mortality has been chosen as a global standard health indicator because it minimizes confounding across countries, it controls for differences in population age structure, and it is independent of other competing causes of mortality. The lower age of 30 years was chosen because that is an age when mortality risk for four chronic diseases starts to rise in most countries. Seventy years of age was chosen as the upper limit of age because at older ages deaths were mostly coded as ill-defined causes and people have other confounding comorbidities that could affect the assignment of cause of death. Furthermore, for this age range 30-70 years, in almost all regions of the world, the majority of deaths can be attributed to the four chronic disease groups (cancer, cardiovascular disease, diabetes, and chronic respiratory disease).[1-2] We focused solely on cancer and calculated overall premature mortality for all cancers and 13 different types of cancers. Premature mortality is the unconditional probability of dying between ages 30 and 70 years from a specific cause. It is calculated utilizing life table methods by 5year age groups and mortality rates from a specific cause of death.[1-3]

Premature Mortality Estimate 2030

For the secondary analysis to benchmark the historical trends with the SDG target, the percentage reduction can be assumed constant for all age-groups and all causes addressed by the NCD target. To achieve the target, we reduced mortality rates from each individual type of cancers and other three major NCDs at the same rate for all 5-year age groups from age 30 to 70 years.

Average Annual Rate of Change in Premature mortality Logarithmic Formula

This formula is conventionally used for health-related SDG indicator monitoring in the World Health Statistics report as below.[2]

R=(ln(pm_t2)-ln(pm_t1))/(t2-t1)

pm=premature mortality
t=year

Categorization Targets to Reach SDG 3.4 Target

1	<0	Very Low
2	≥0-0.84	Low
3	≥ 0.85-1.1	High
4	>1.2	Very High

References

1. World Health Organization. Global Health Observatory, premature mortality from non-communicable disease.[online]. [Accessed 29 June 2023]. Available from: https://www.who.int/data/gho/indicator-metadata-registry/imr-details/3411
2. World Health Organization. WHO methods and data sources for country-level causes of death 2000-2019. Department of Data and Analytics (DNA). Division of Data, Analytics and Delivery for Impact. [online].WHO Geneva December 2020 [Accessed 29 June 2023]. Available from https://www.who.int/docs/default-source/gho-documents/global-health-estimates/ghe2019_cod_methods.pdf?sfvrsn=37bcfacc_5
3. World Health Organization. (2014). Global status report on non-communicable diseases. [online]. 2014 [Accessed 29 June 2023] Available from: https://apps.who.int/iris/handle/10665/148114

Figures by Sex, World Bank Income Level, and WHO Region

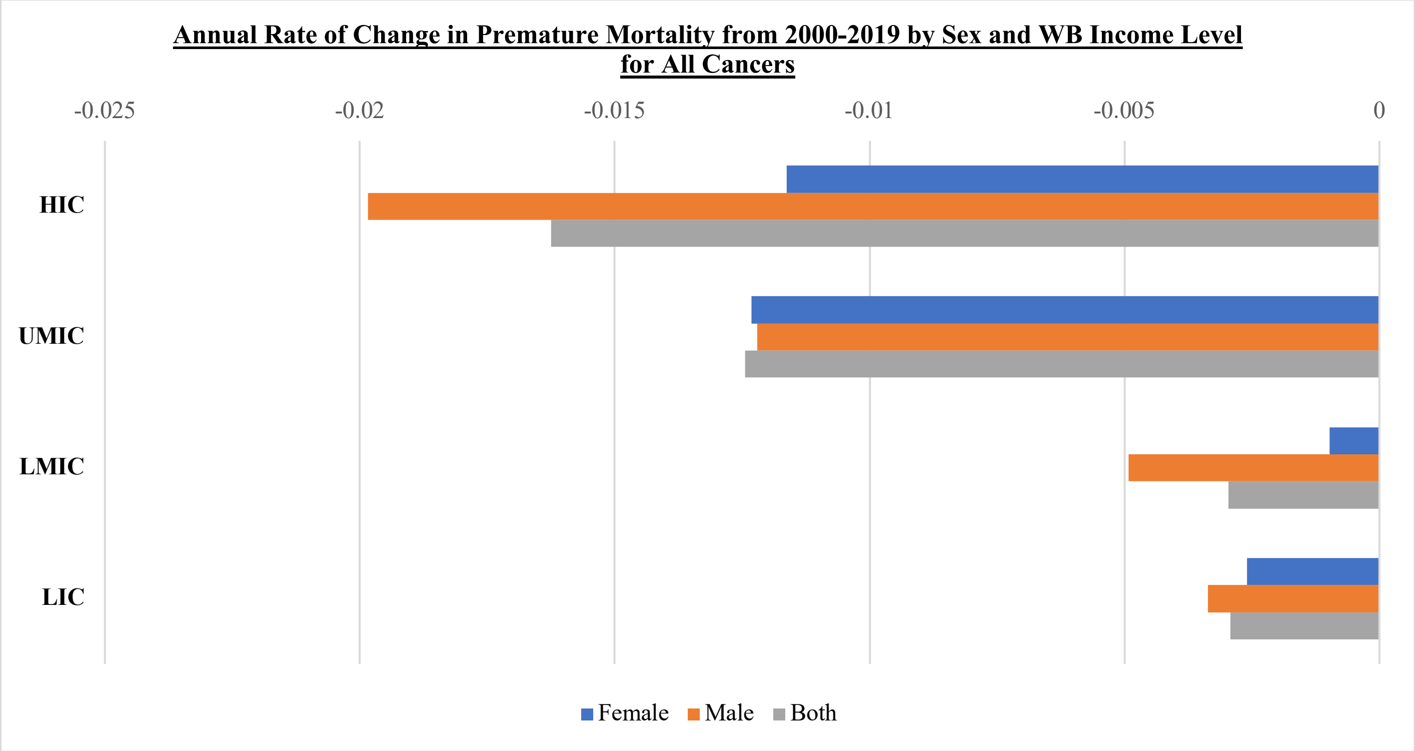

Figure 4 Trends in the annual rate of change in premature mortality from 2000-2019 for all cancers by sex and world bank income level

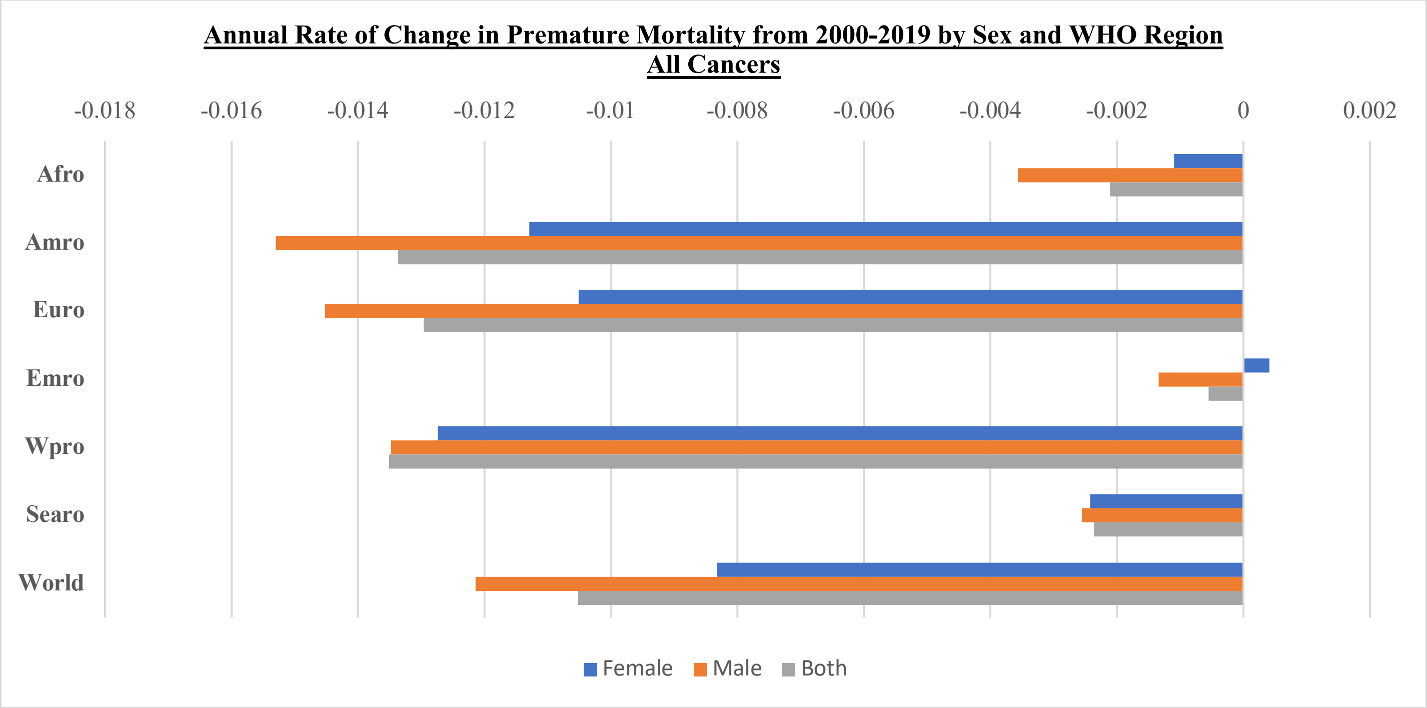

Figure 5: Trends in the annual rate of change in premature mortality from 2000-2019 for all cancers by sex and world health organization region

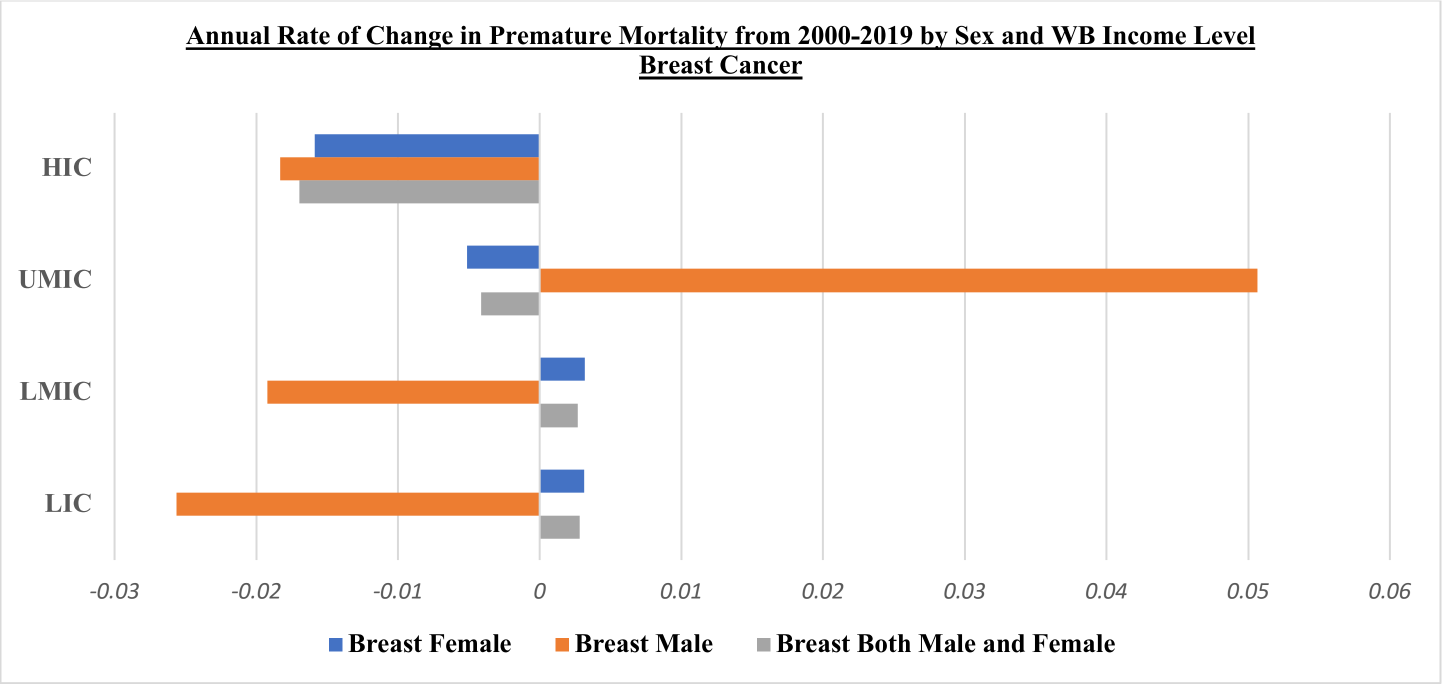

Figure 6: Trends in the annual rate of change in premature mortality from 2000-2019 for breast cancer by sex and world bank income level

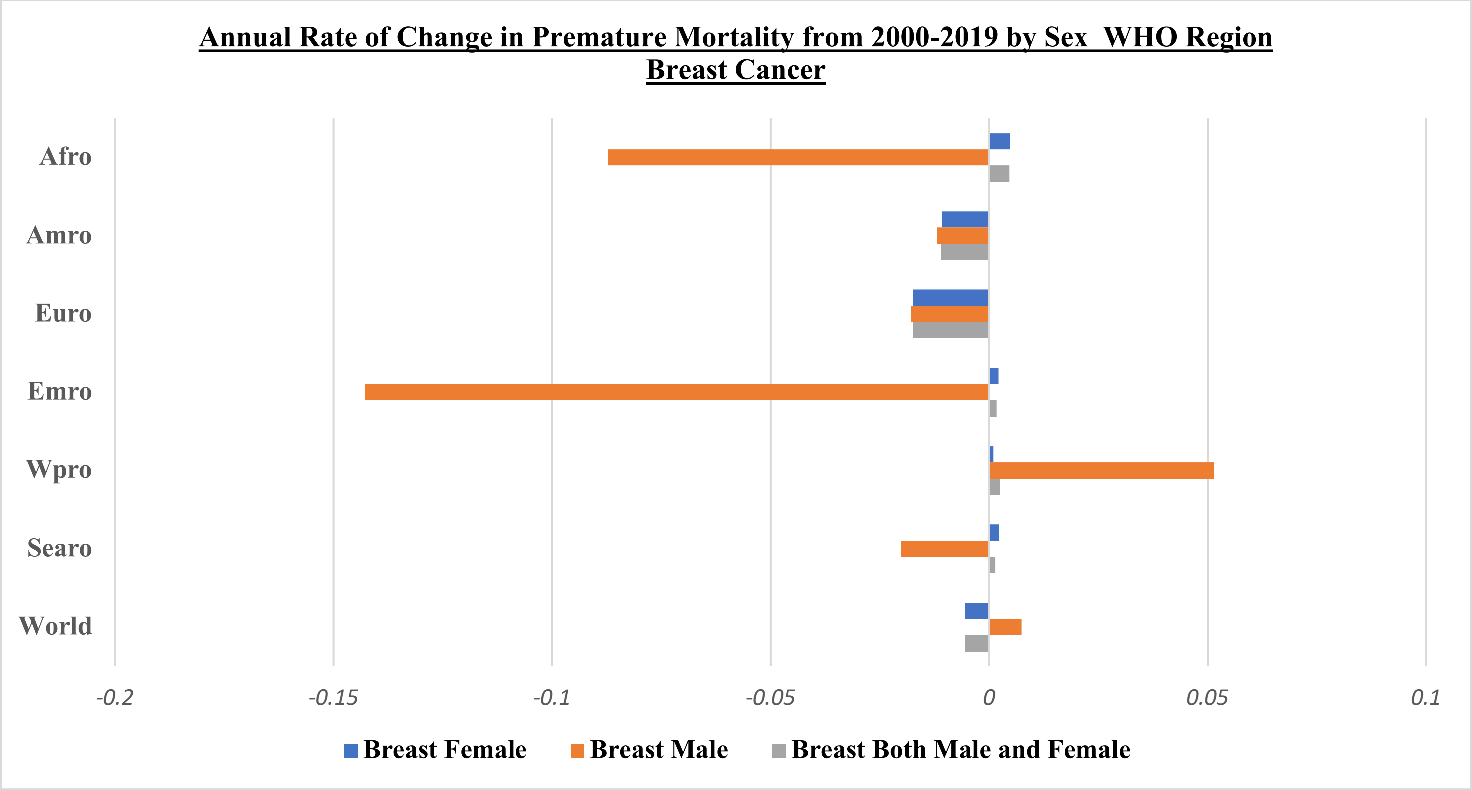

Figure 7: Trends in the annual rate of change in premature mortality from 2000-2019 for breast cancer by sex and world health organization region

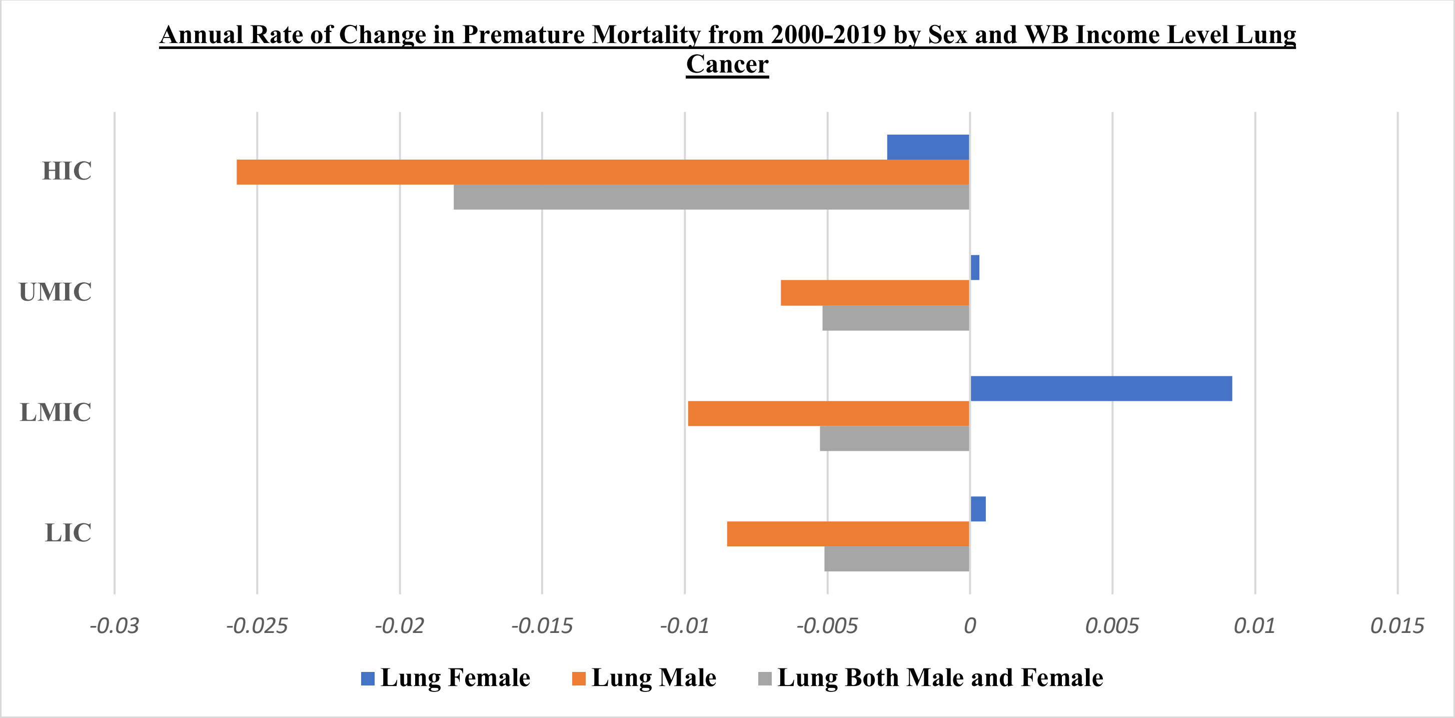

Figure 8: Trends in the annual rate of change in premature mortality from 2000-2019 for lung cancer by sex and world bank income level

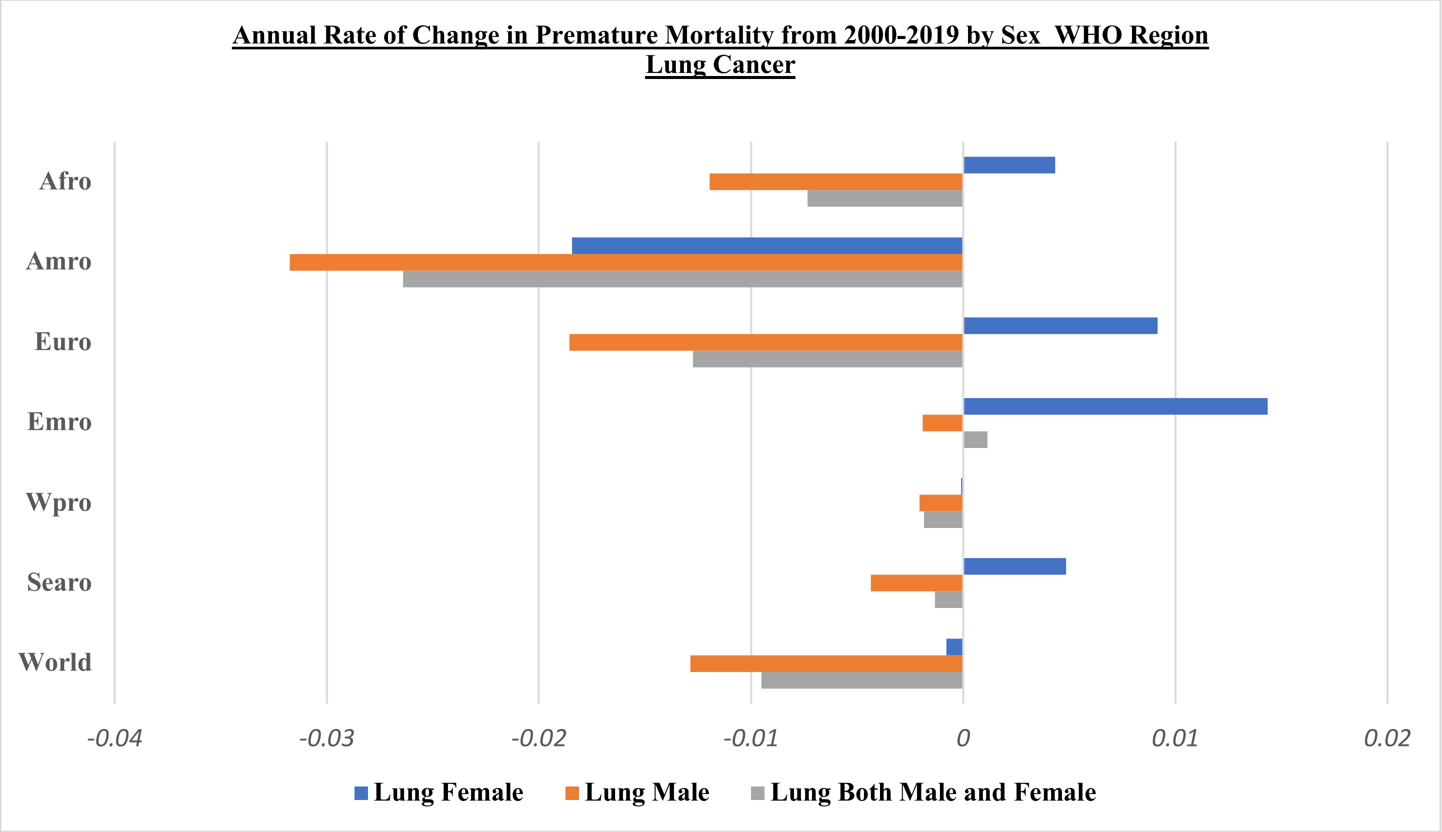

Figure 9: Trends in the annual rate of change in premature mortality from 2000-2019 for lung cancer by sex and world health organization region

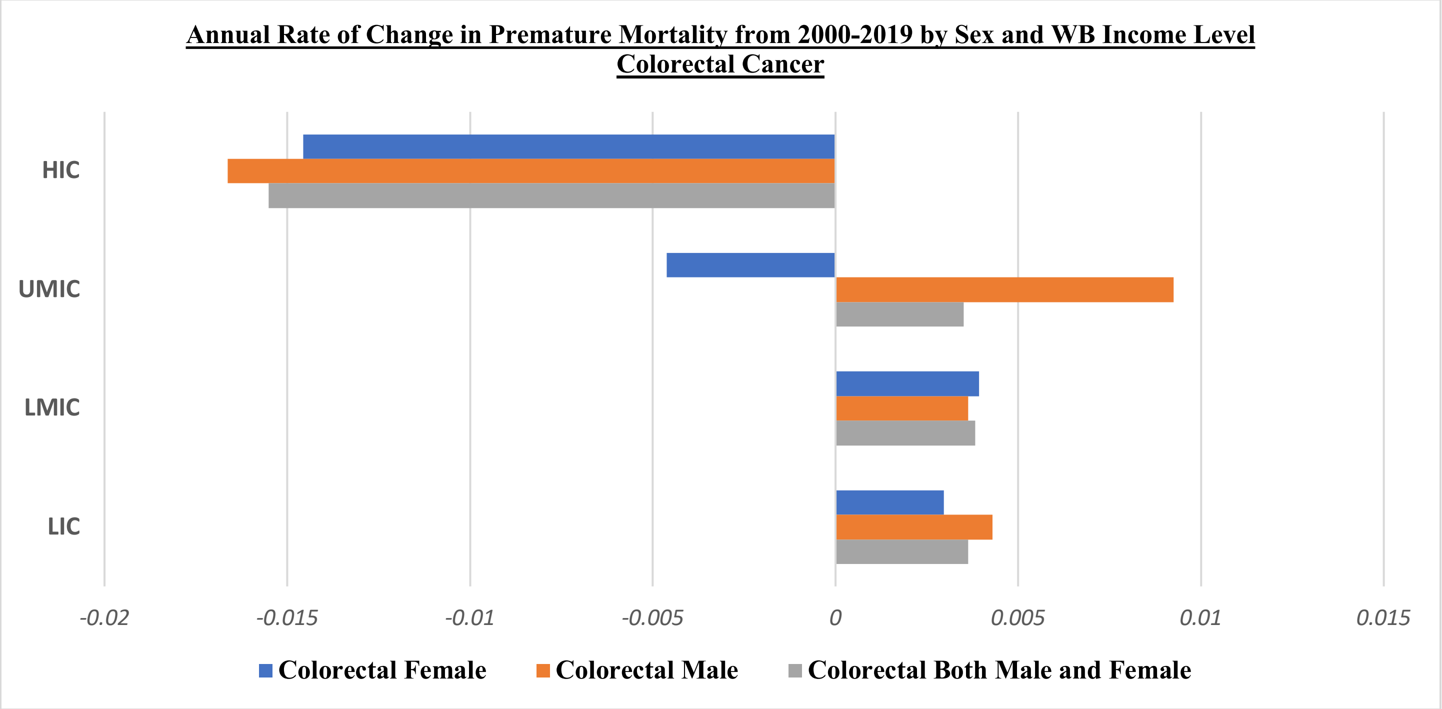

Figure 10: Trends in the annual rate of change in premature mortality from 2000-2019 for colorectal cancer by sex and world bank income level

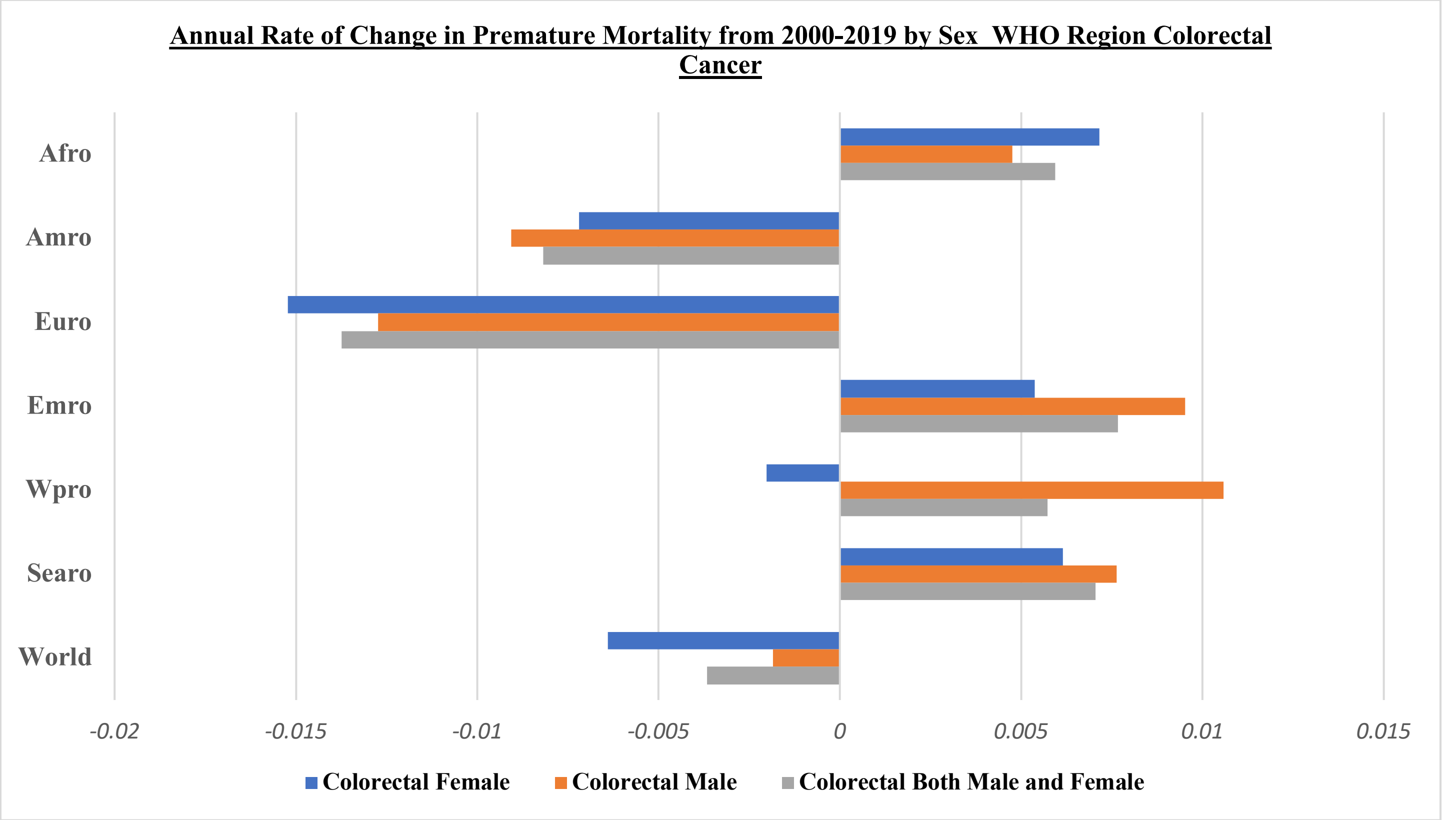

Figure 11: Trends in the annual rate of change in premature mortality from 2000-2019 for colorectal cancer by sex and world health organization region

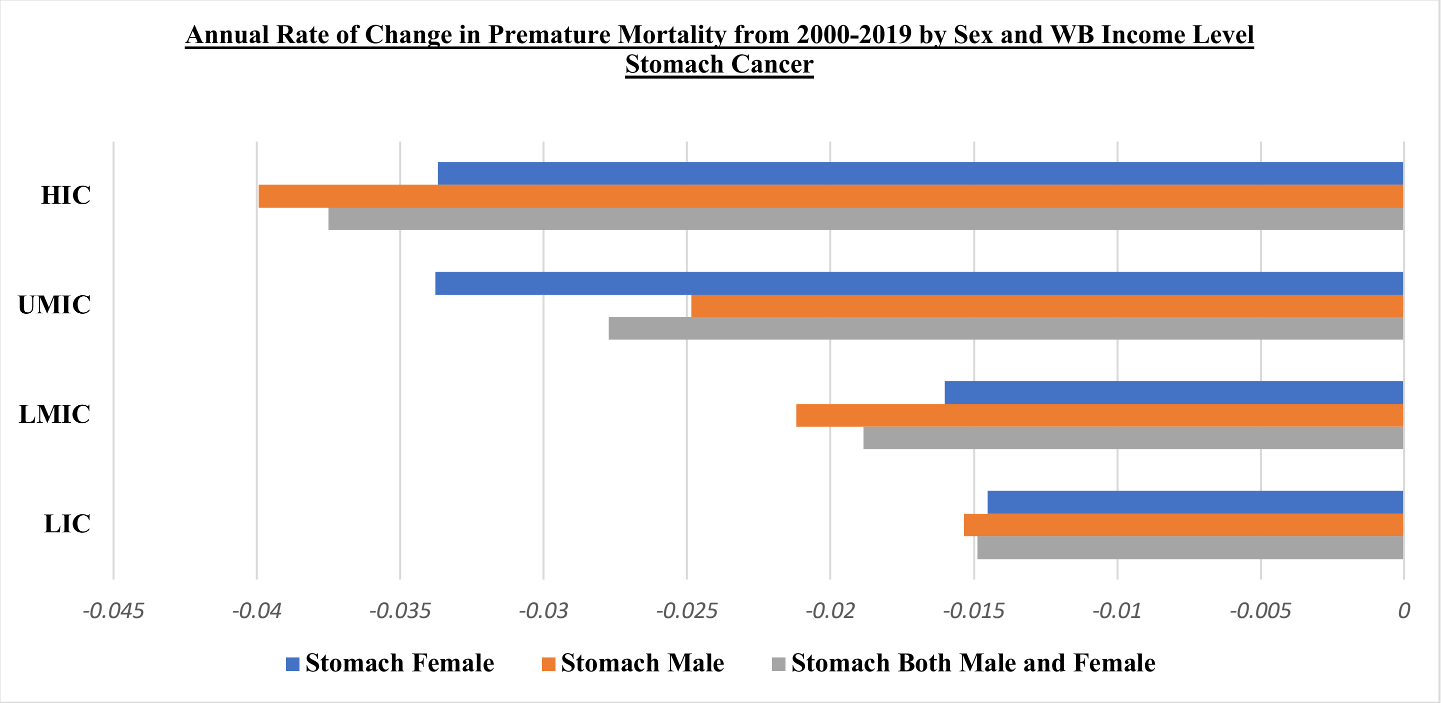

Figure 12: Trends in the annual rate of change in premature mortality from 2000-2019 for stomach cancer by sex and world health organization region

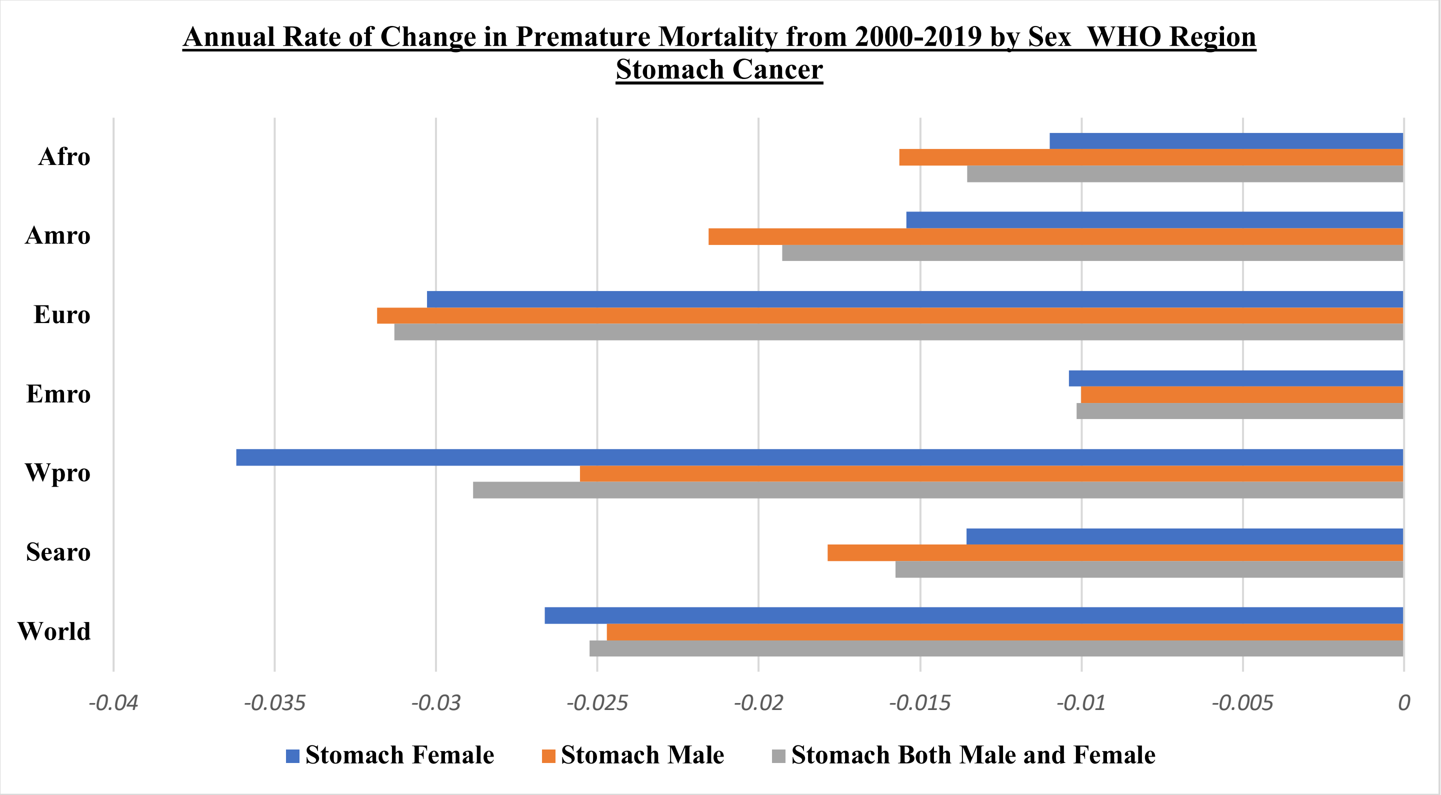

Figure 13: Trends in the annual rate of change in premature mortality from 2000-2019 for stomach cancer by sex and world health organization region

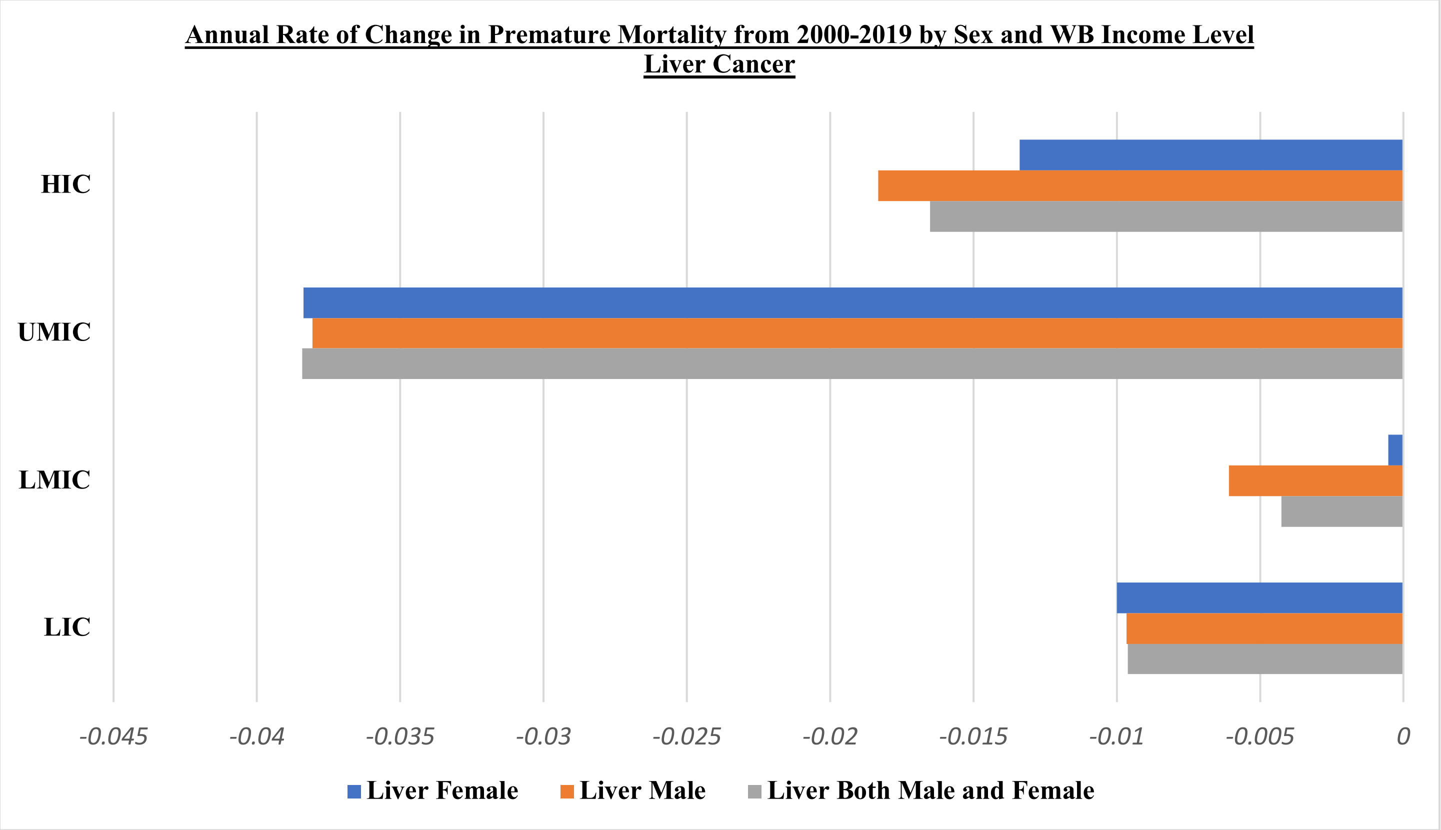

Figure 14: Trends in the annual rate of change in premature mortality from 2000-2019 for liver cancer by sex and world bank income level

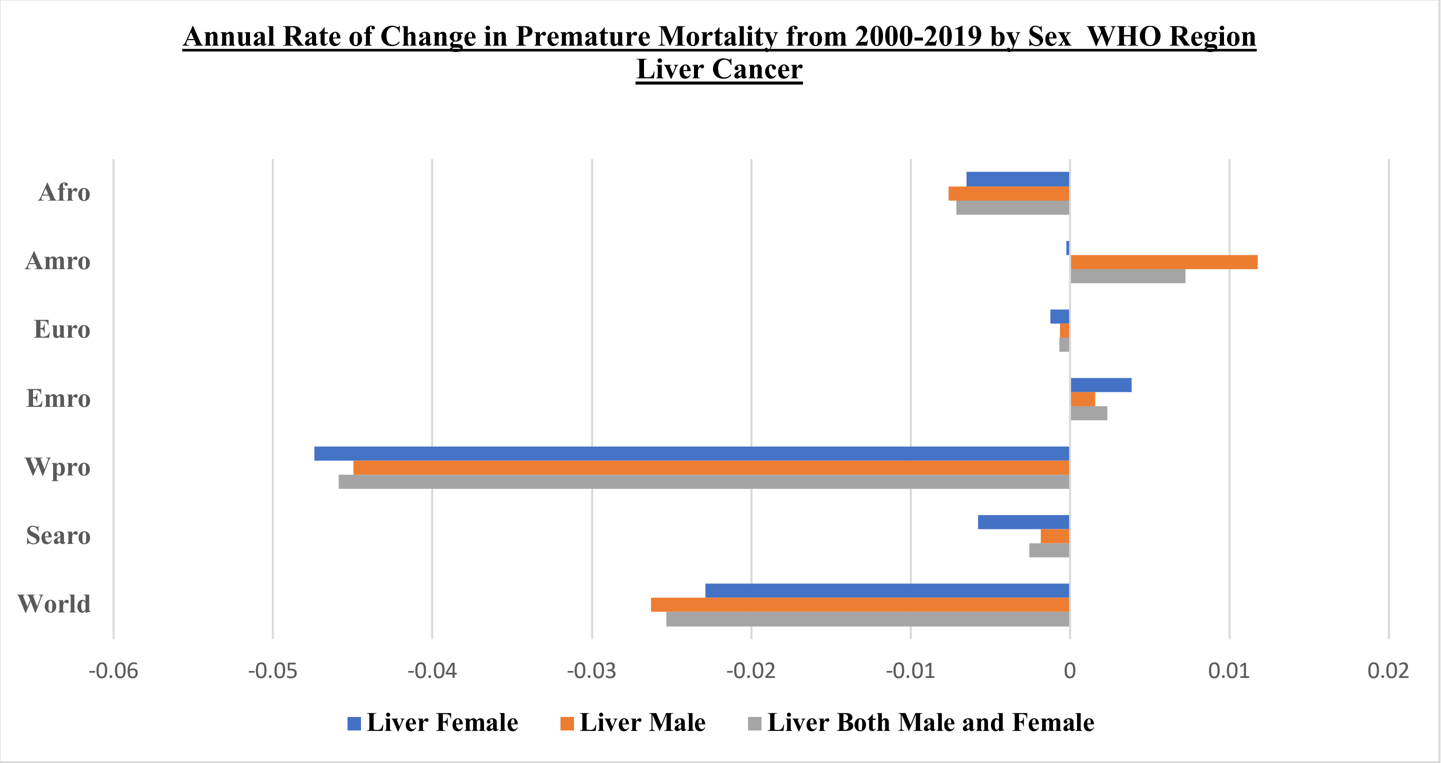

Figure 15: Trends in the annual rate of change in premature mortality from 2000-2019 for liver cancer by sex and world health organization region

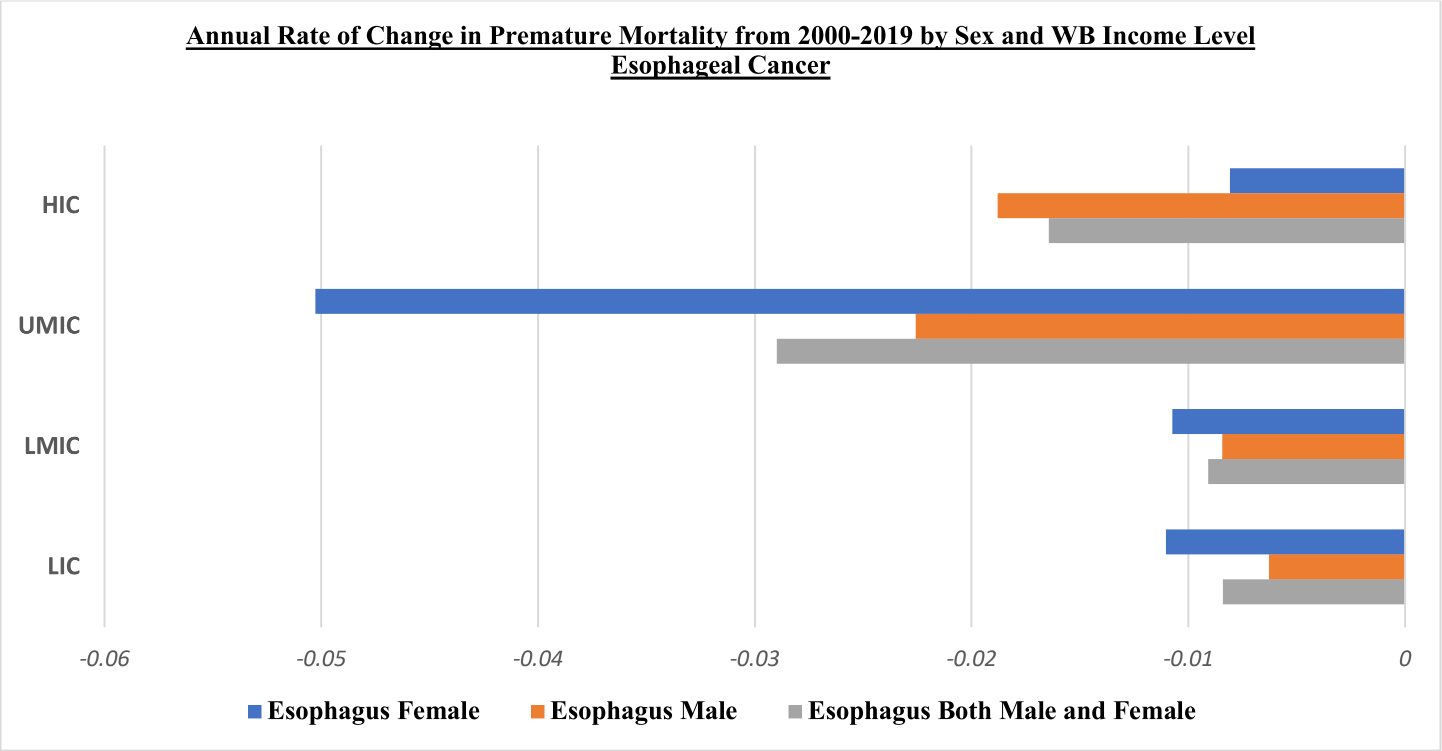

Figure 16: Trends in the annual rate of change in premature mortality from 2000-2019 for esophageal cancer by sex and world bank income level

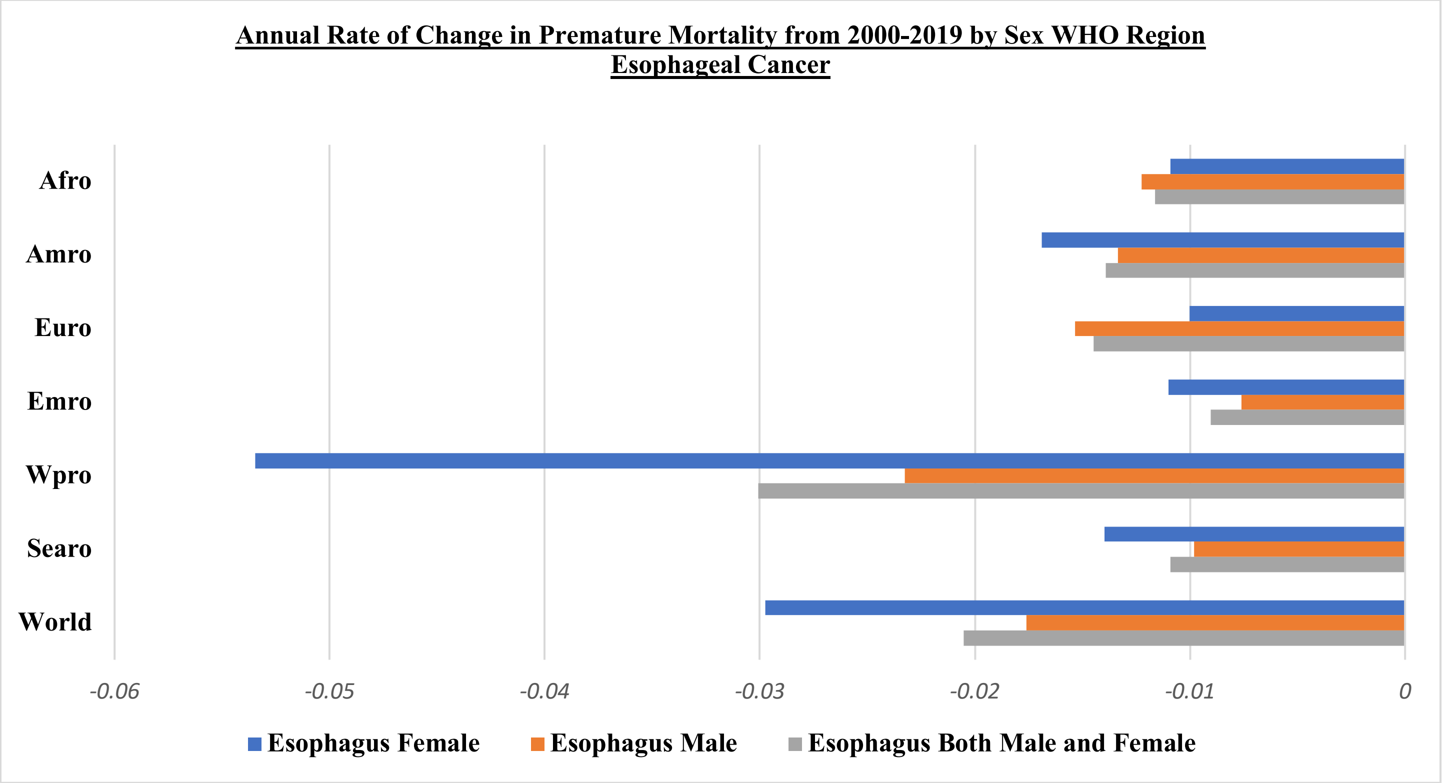

Figure 17: Trends in the annual rate of change in premature mortality from 2000-2019 for esophageal cancer by sex and world health organization region

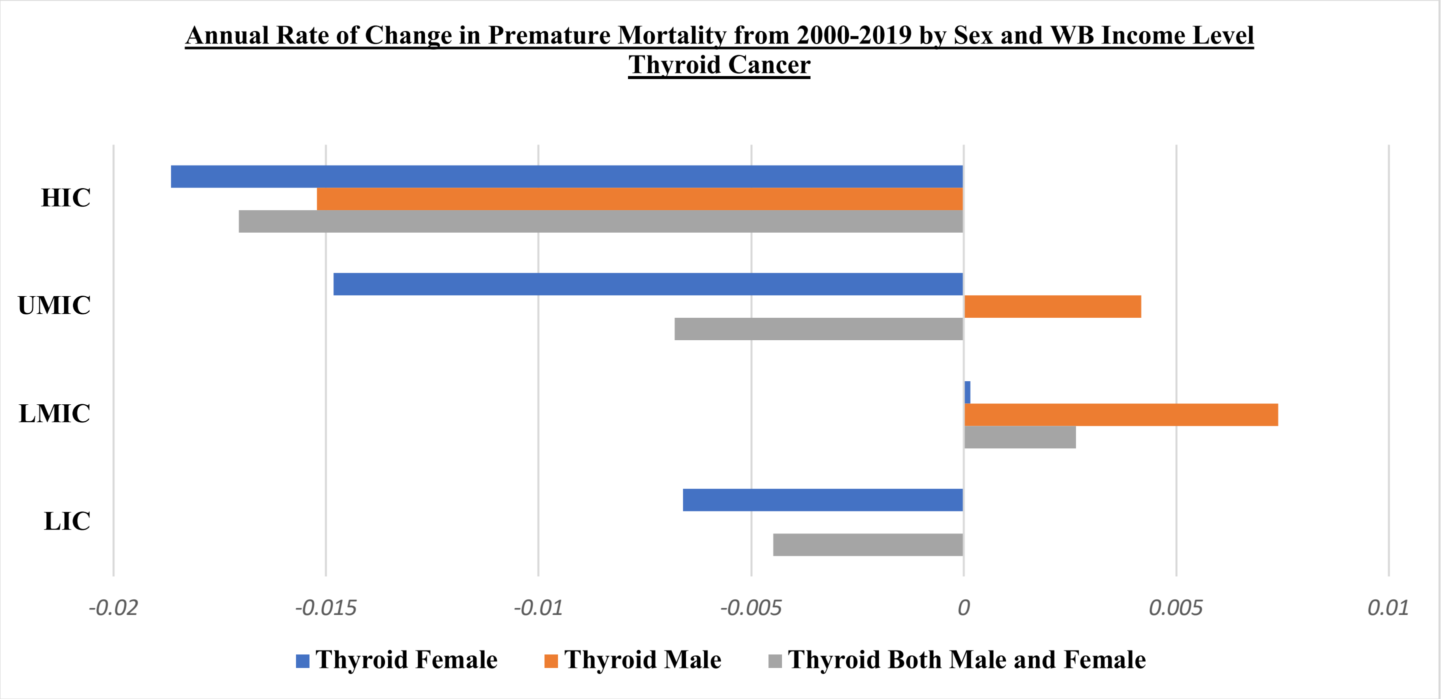

Figure 18: Trends in the annual rate of change in premature mortality from 2000-2019 for thyroid cancer by sex and world bank income level

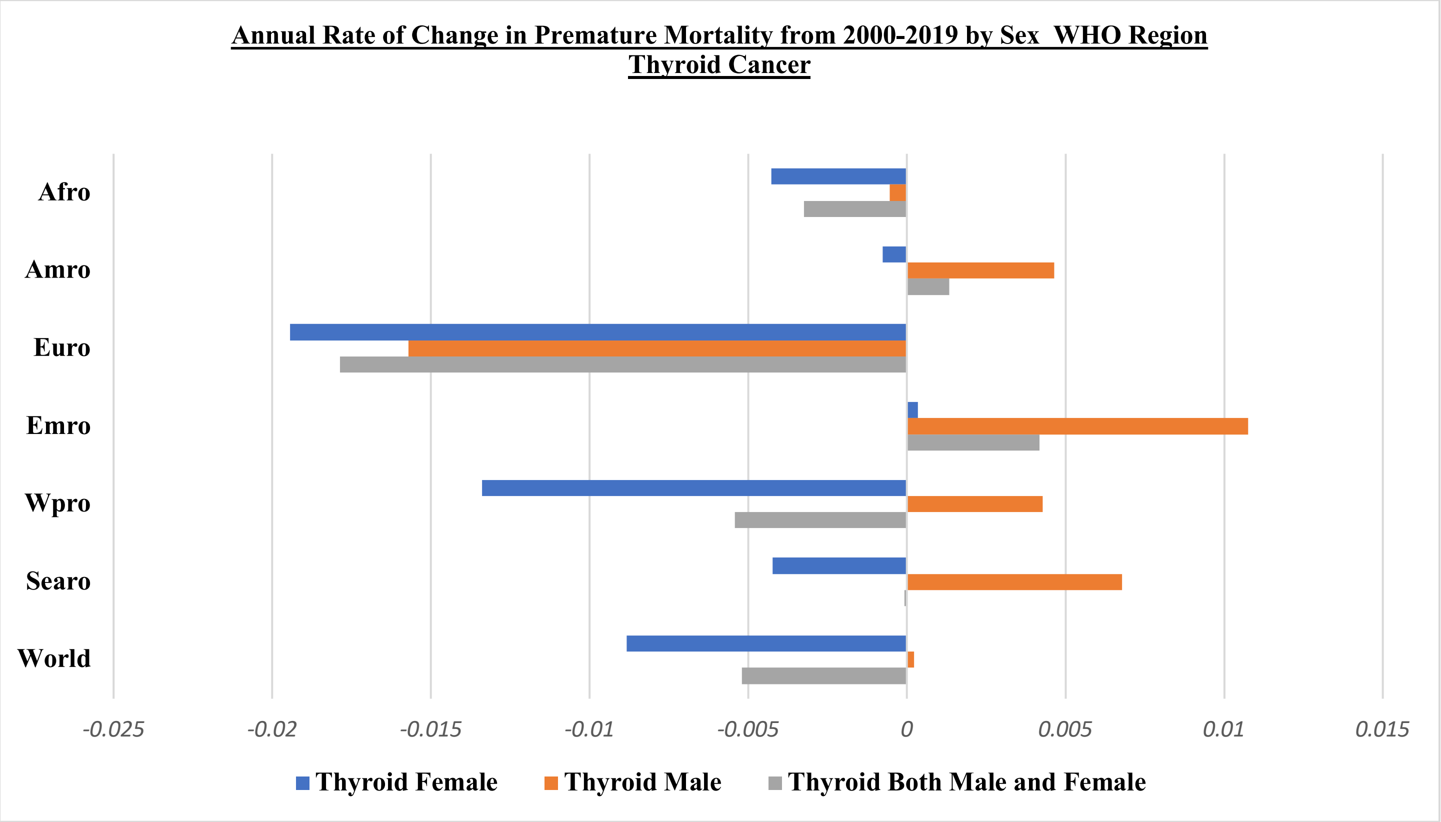

Figure 19: Trends in the annual rate of change in premature mortality from 2000-2019 for thyroid cancer by sex and world health organization

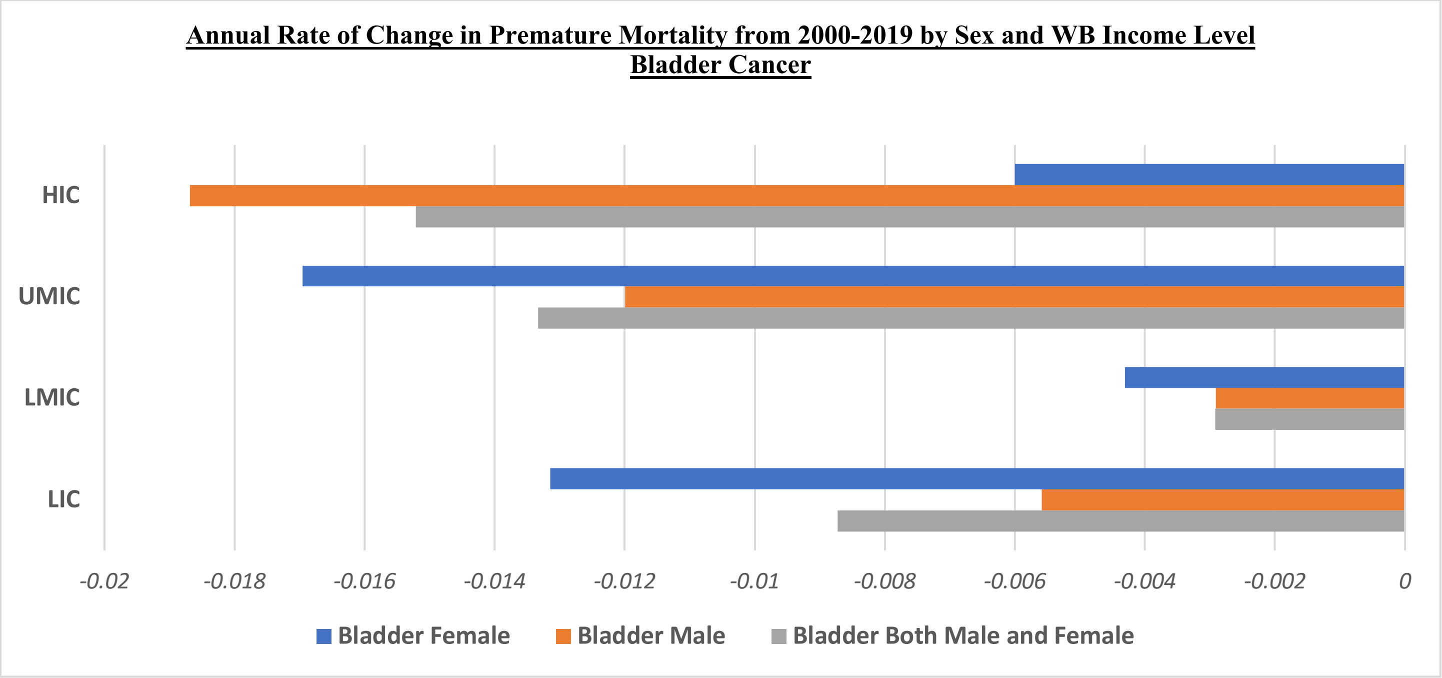

Figure 20: Trends in the annual rate of change in premature mortality from 2000-2019 for bladder cancer by sex and world bank income level

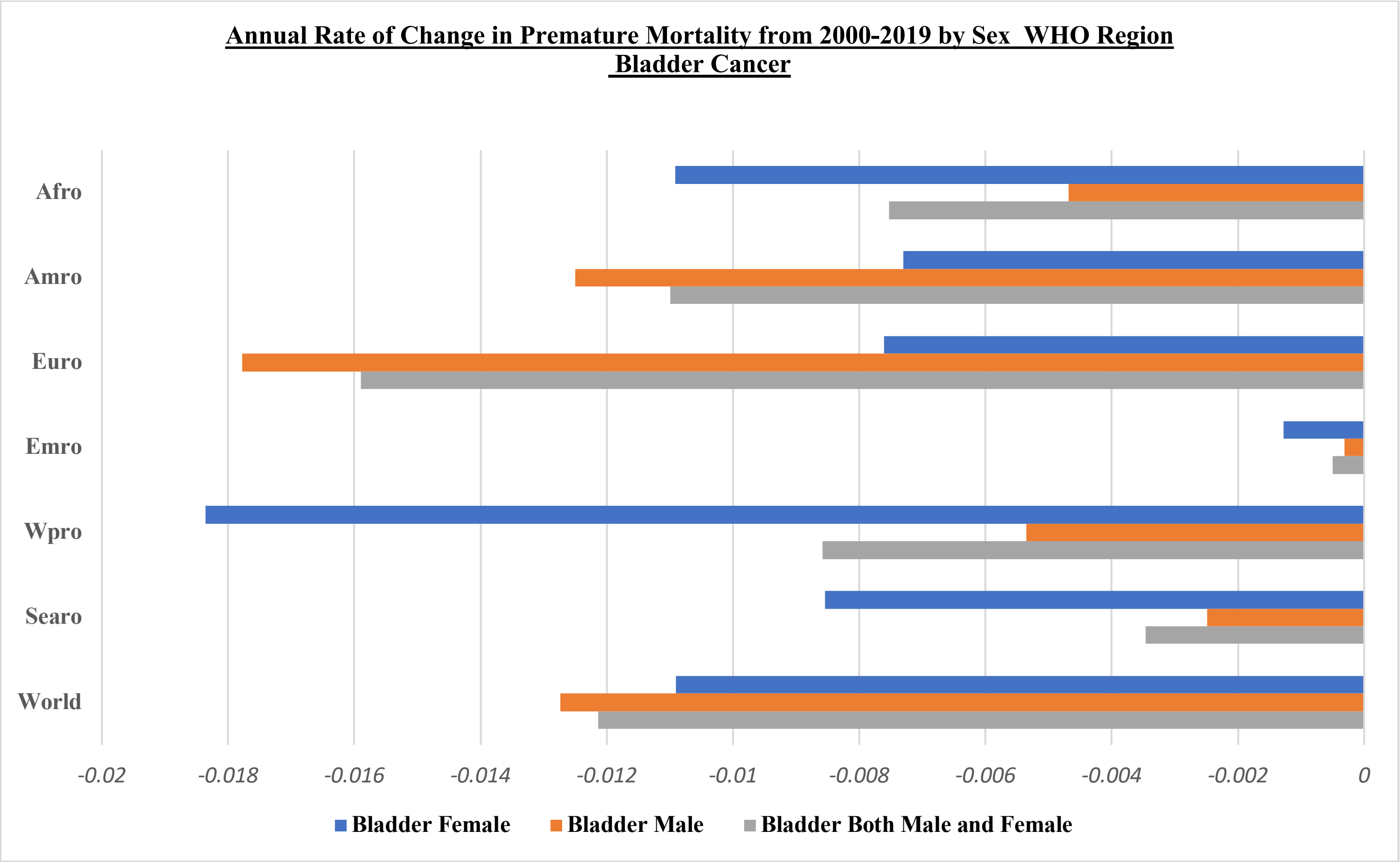

Figure 21: Trends in the annual rate of change in premature mortality from 2000-2019 for bladder cancer by sex and world health organization region

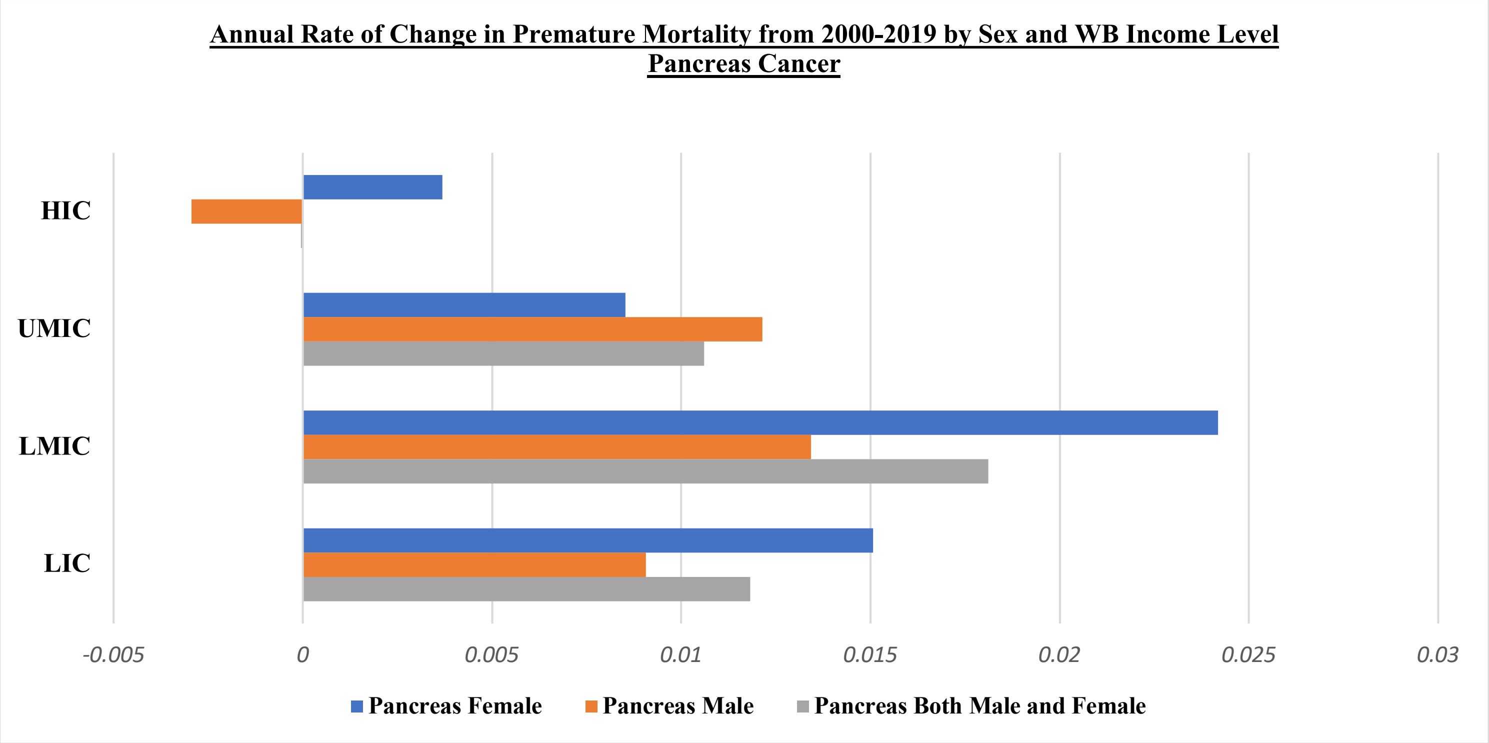

Figure 22: Trends in the annual rate of change in premature mortality from 2000-2019 for pancreas cancer by sex and world bank income level

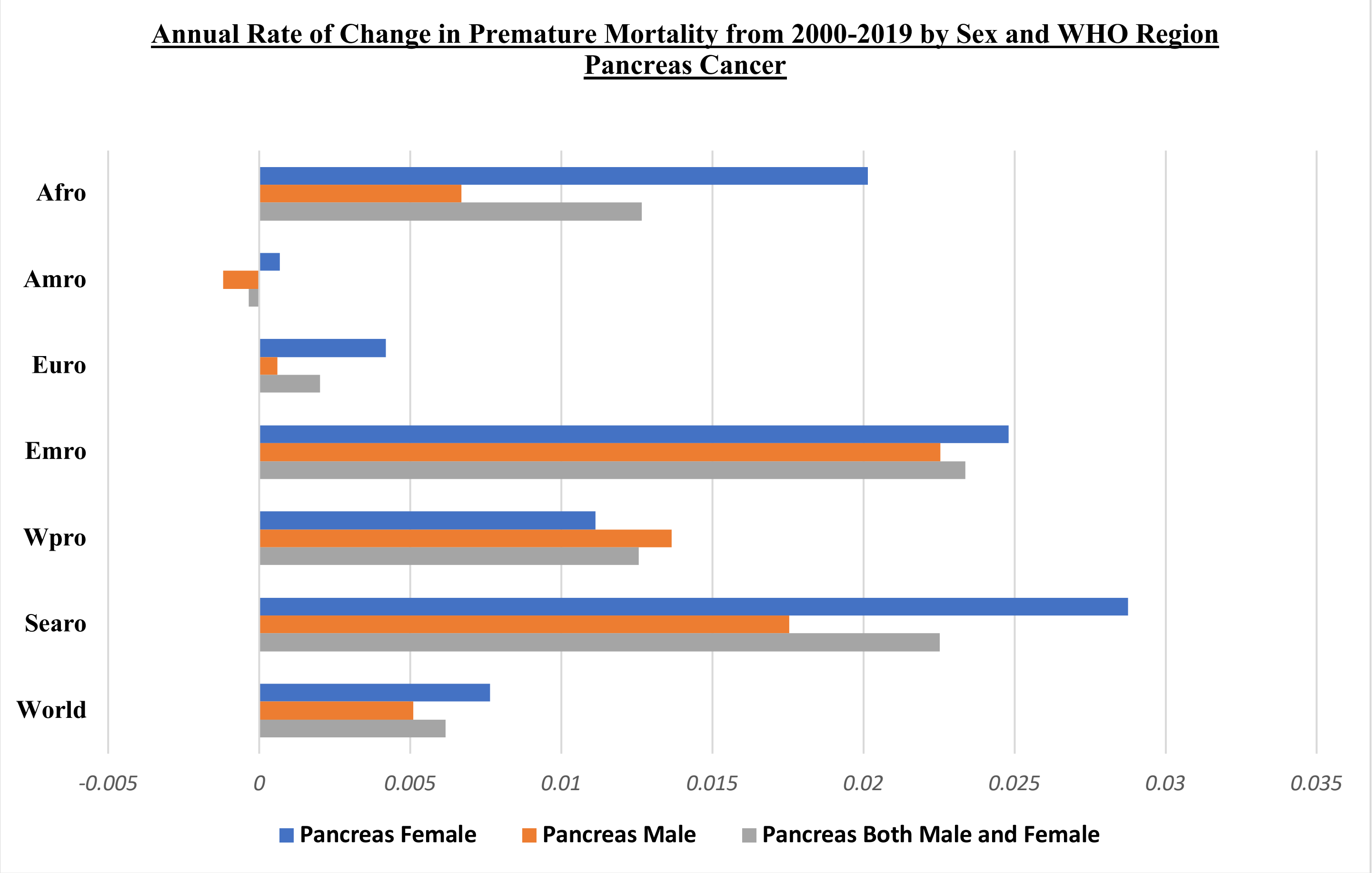

Figure 23: Trends in annual rate of change in premature mortality from 2000-2019 for pancreas cancer by sex and world health organization region

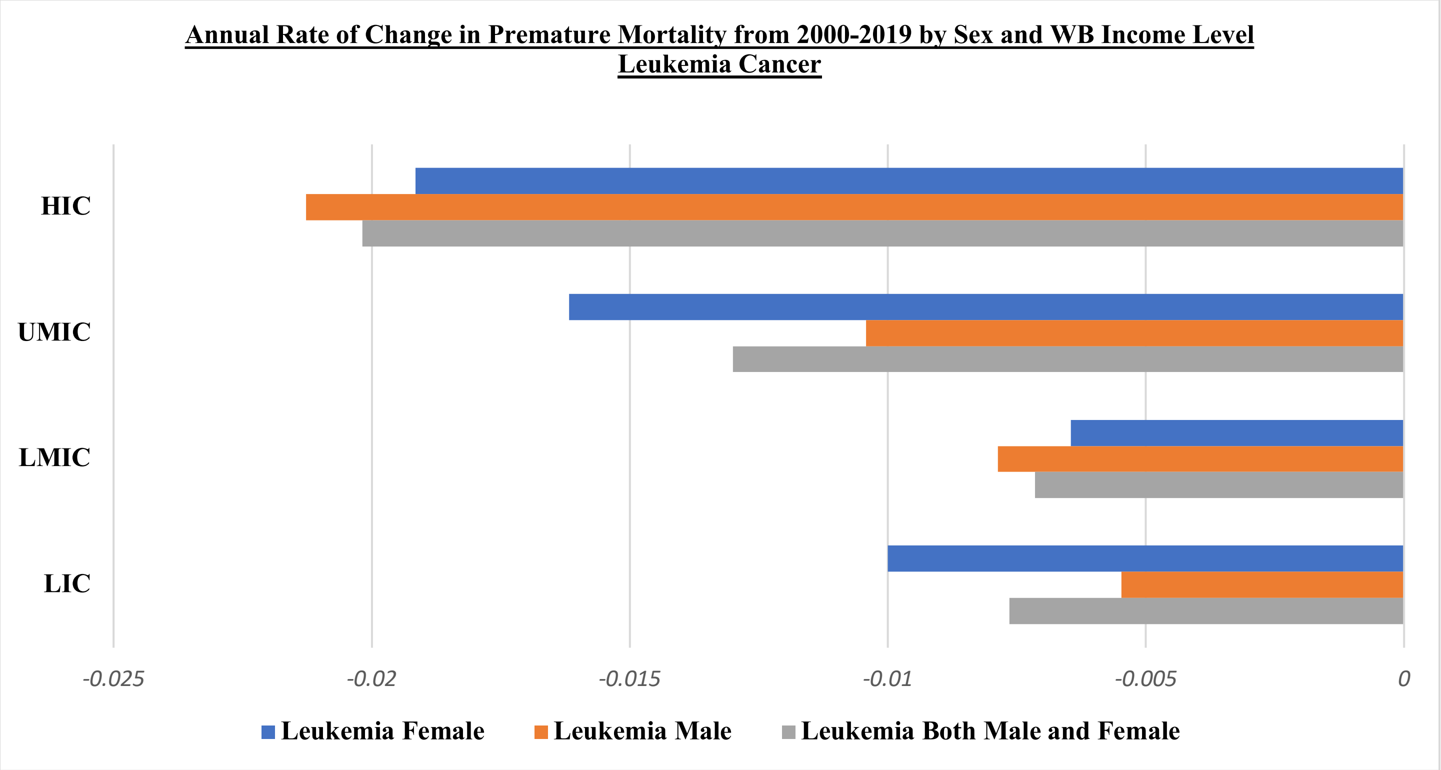

Figure 24: Trends in the annual rate of change in premature mortality from 2000-2019 for leukemia cancer by sex and world bank income level

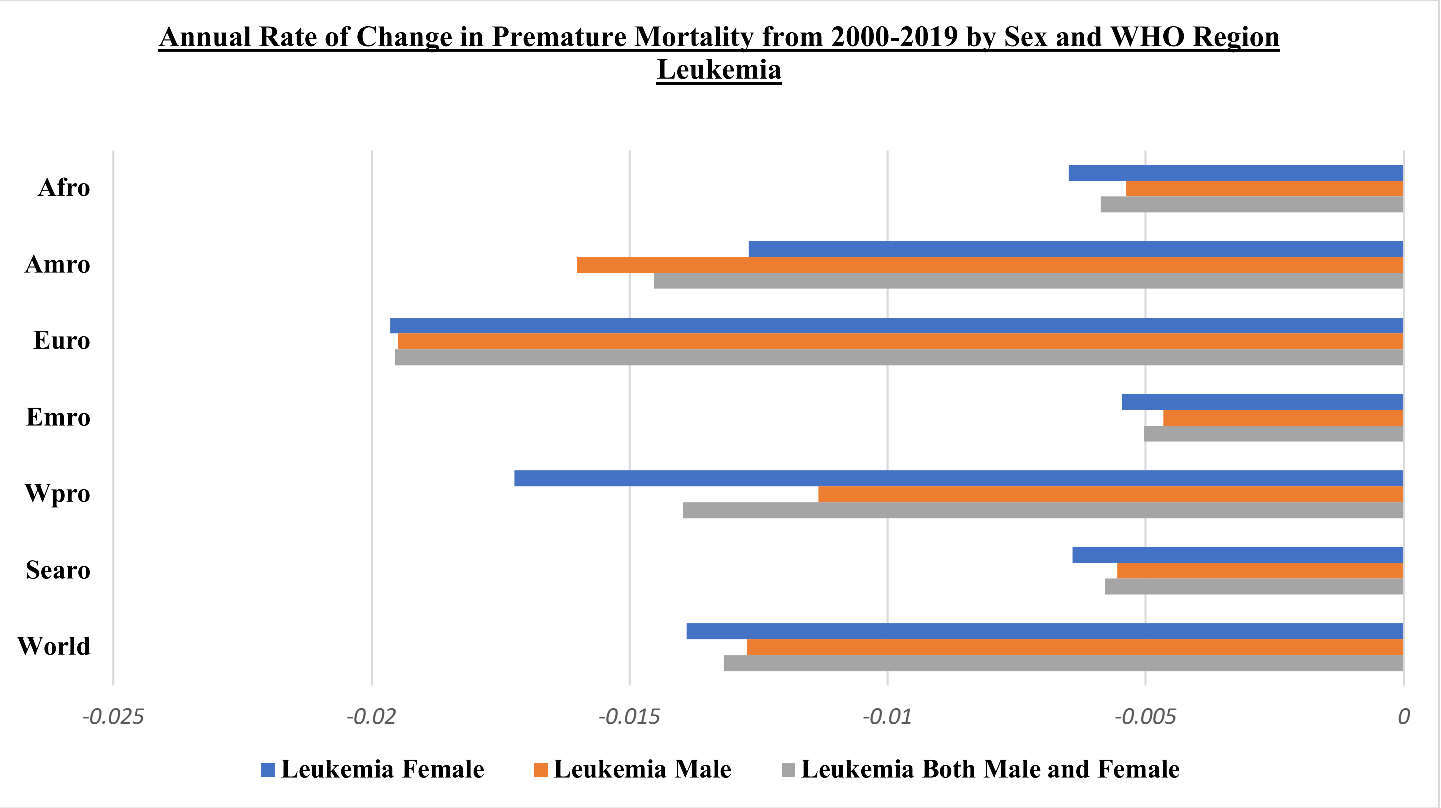

Figure 25: Trends in the annual rate of change in premature mortality from 2000-2019 for leukemia cancer by sex and world health organization region

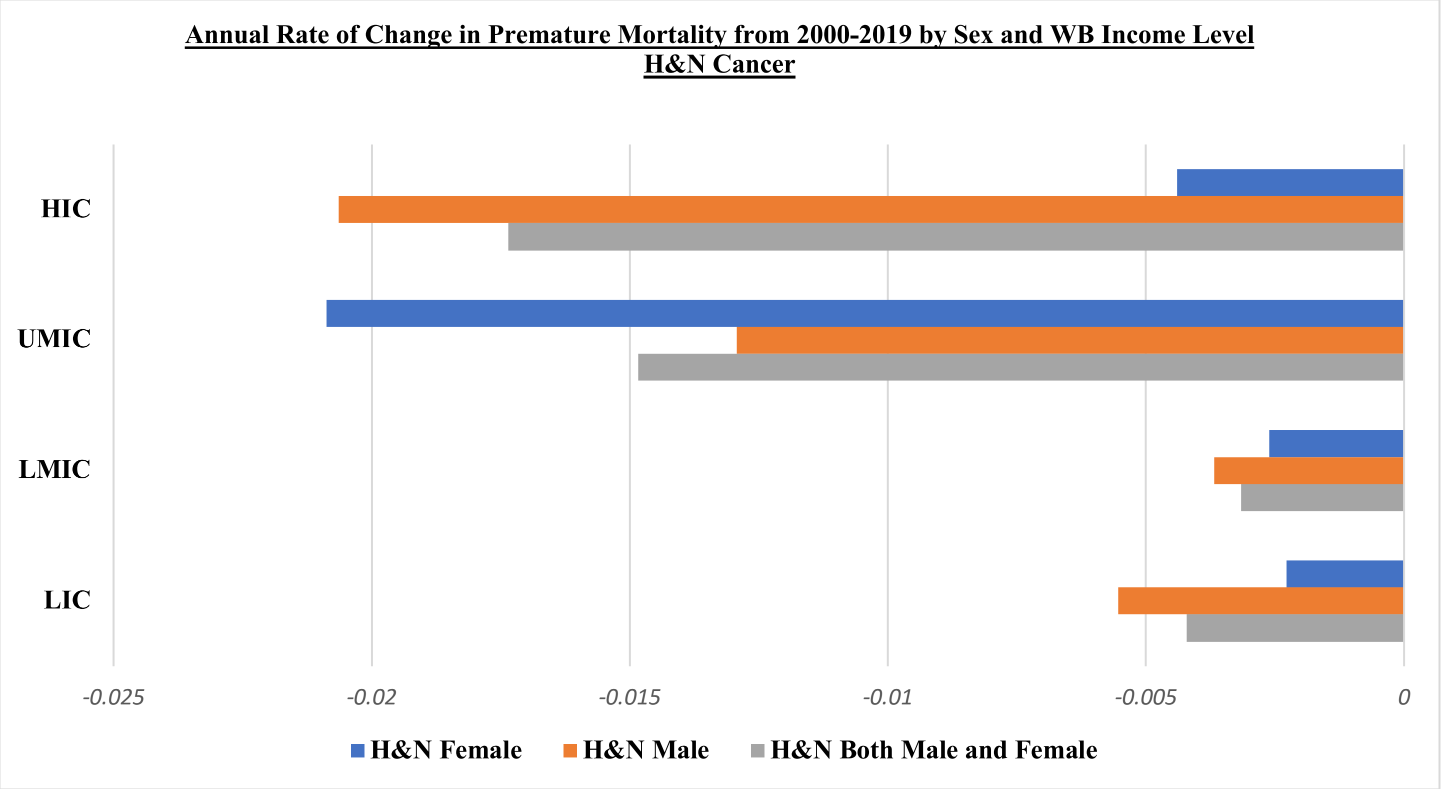

Figure 26: Trends in the annual rate of change in premature mortality from 2000-2019 for head and neck cancer by sex and world bank income level

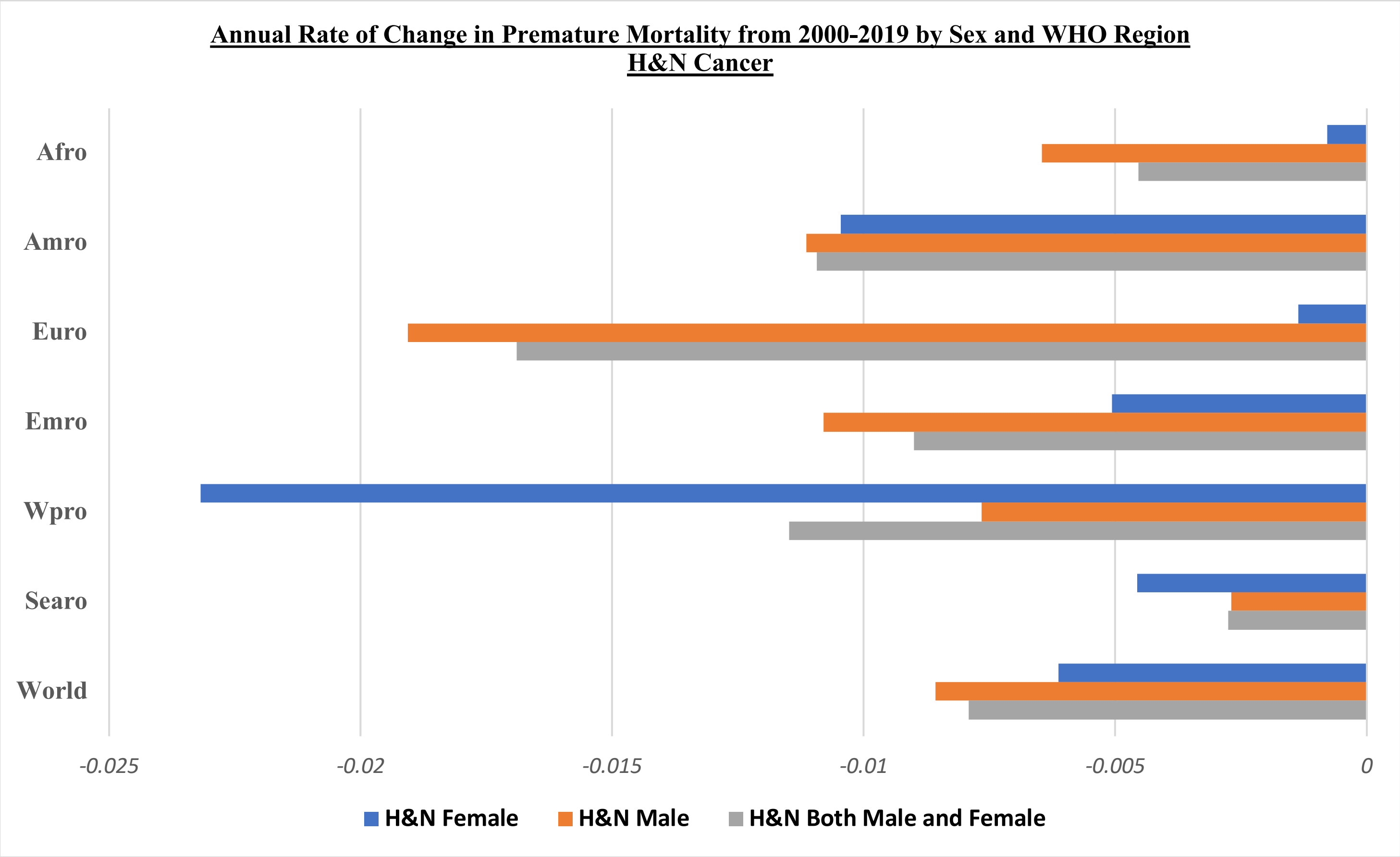

Figure 27: Trends in the annual rate of change in premature mortality from 2000-2019 for head and neck cancer by sex and world health organization region
